# Modulating the surface potential of microspheres by phase transition in strontium doped barium titanate to restore the electric microenvironment for bone regeneration

**DOI:** 10.3389/fbioe.2022.988300

**Published:** 2022-08-30

**Authors:** Peng Wang, Xiaosong Zhou, Caili Lv, Yu Wang, Zongliang Wang, Liqiang Wang, Yongzhan Zhu, Min Guo, Peibiao Zhang

**Affiliations:** ^1^ Key Laboratory of Polymer Ecomaterials, Changchun Institute of Applied Chemistry, Chinese Academy of Sciences, Changchun, China; ^2^ School of Applied Chemistry and Engineering, University of Science and Technology of China, Hefei, China; ^3^ Department of Ophthalmology, Third Medical Center, Chinese PLA General Hospital, Beijing, China; ^4^ 8th Department of Orthopaedics, Foshan Hospital of Traditional Chinese Medicine, Foshan, China

**Keywords:** microsphere, surface potential, dielectric properties, bone regeneration, doped, barium titanate

## Abstract

The endogenous electrical potential generated by native bone and periosteum plays a key role in maintaining bone mass and quality. Inspired by the electrical properties of bone, different negative surface potentials are built on microspheres to restore electric microenvironment for powerful bone regeneration, which was prepared by the combination of strontium-doped barium titanate (Sr-BTO) nanoparticles and poly (lactic-co-glycolic acid) (PLGA) with high electrostatic voltage field (HEV). The surface potential was modulated through regulating the phase composition of nanoparticles in microspheres by the doping amount of strontium ion (Sr^2+^). As a result, the 0.1Sr-BTO/PLGA group shows the lowest surface potential and its relative permittivity is closer to natural bone. As expected, the 0.1Sr-BTO/PLGA microspheres performed cytocompatibility, osteogenic activity *in vitro* and enhance bone regeneration *in vivo*. Furthermore, the potential mechanism of Sr-BTO/PLGA microspheres to promote osteogenic differentiation was further explored. The lower surface potential generated on Sr-BTO/PLGA microspheres regulates cell membrane potential and leads to an increase in the intracellular calcium ion (Ca^2+^) concentration, which could activate the Calcineurin (CaN)/Nuclear factor of activated T-cells (NFAT) signaling pathway to promote osteogenic differentiation. This study established an effective method to modulate the surface potential, which provides a prospective exploration for electrical stimulation therapy. The 0.1Sr-BTO/PLGA microsphere with lower surface potential and bone-matched dielectric constant is expected to have great potential in the field of bone regeneration.

## 1 Introduction

Bone defects caused by trauma, tumor resection, and vascular injuries may cause life-long disability of patient (Gan et al., 2021). At present, autologous bone grafts and allogeneic bone grafts as the most common method for treating bone defects still face many problems, such as limited bone sources, poor geometrical suitability for targeted sites, and the possibility of carrying pathogens (Wang et al., 2021). Hence, it is imperative to develop artificial bone substitutes regulating to achieve rapid and efficient healing of defective bones.

To address these limitations, injectable microspheres are attracting more attention because they are suitable for perfectly filling irregularly shaped defects (Fang et al., 2019; Wei et al., 2019; Yan et al., 2021). At the same time, large-size polymer microspheres can be stacked in a three-dimensional manner with large porosity, which is conducive to nutrient exchange and cell migration. However, some drawbacks still need to be solved, such as low cell loading capacity, poor cell survival rate and osteogenic activity. Imitating the chemical composition of natural bones and using osteogenic factors are generally considered to be effective and traditional means to improve the osteogenic activity of materials (Kim et al., 2021; Ding et al., 2022). For example, inorganic materials such as hydroxyapatite or β-tricalcium phosphate, and growth factors such as bone morphogenetic protein 2 (BMP-2) or transforming growth factor-β 1 (TGF-β1) were introduced into materials to improve osteogenic activity (Sun et al., 2021; Xu et al., 2021; Li et al., 2022). Alongside the abovementioned biochemical cues, constructing an electrical microenvironment is emerging as an effectively strategy to accelerate bone regeneration owing to their higher safety, lower cost, and tunable localized properties (Kong et al., 2021). In the human body, various types of tissues present electrically sensitive properties such as the nervous system, heart, bone, muscle, and skin (Shamos and Lavine, 1967; Ning et al., 2018). Previous related studies have also confirmed that electrical stimulation plays an important role in regulating cell differentiation and tissue regeneration (Khare et al., 2020). Particularly in bone injury, the behavioral guidance of cells is influenced by responsive bioelectric cues (Kaliannagounder et al., 2021). As far back as 1957, Yasuda and Fukada first discovered the piezoelectric property of natural bone (Fukada and Yasuda, 1957). Furthermore, studies have found that the electrical properties of bone are a key factor in maintaining bone volume and quality (Bassett et al., 1964; Trumbore et al., 1980). Inspired by the electrical properties of bones, constructing an electrical microenvironment on the surface of microspheres may be an effective solution to achieve the purpose of enhancing the biological activity of the microspheres and bone regeneration.

Barium titanate (BTO) as a traditional electroactive nanoparticle possess good piezoelectric performance, high dielectric constant and capability of spontaneous polarization (Sharma et al., 1999). Importantly, BTO exhibits high biocompatibility and fast metabolic rates to meet the critical requirements of the biomedical field (Li et al., 2017). In recent years, BTO has gradually been applied in bone tissue engineering research. For example, Bo Fan et al. demonstrated that BTO nanoparticles as a modified layer of the scaffold can well promote osteogenic differentiation (Fan et al., 2020). However, it is still a challenge to regulate the electrical activity of barium titanate to be more suitable for bone regeneration. Inorganic ion doping is an effective means to control the electrical properties of BTO. Some bioactive elements have aroused great attention since they are incorporated into composites to improve their osteogenesis and angiogenesis properties, such as magnesium (Mg), calcium (Ca), strontium (Sr), cobalt (Co), lithium (Li), copper (Cu), and zirconium (Zr) (Naruphontjirakul et al., 2018). Among them, Sr and Ba are close to each other in the periodic table and have similar chemical properties and ionic radii. In addition, Sr^2+^ can stimulate the bone-forming function of osteoblasts and inhibit the bone resorbing function of osteoclasts (Lin et al., 2013; Zhang et al., 2018). Based on the above factors, Sr^2+^ is our preferred element.

**SCHEME 1:**
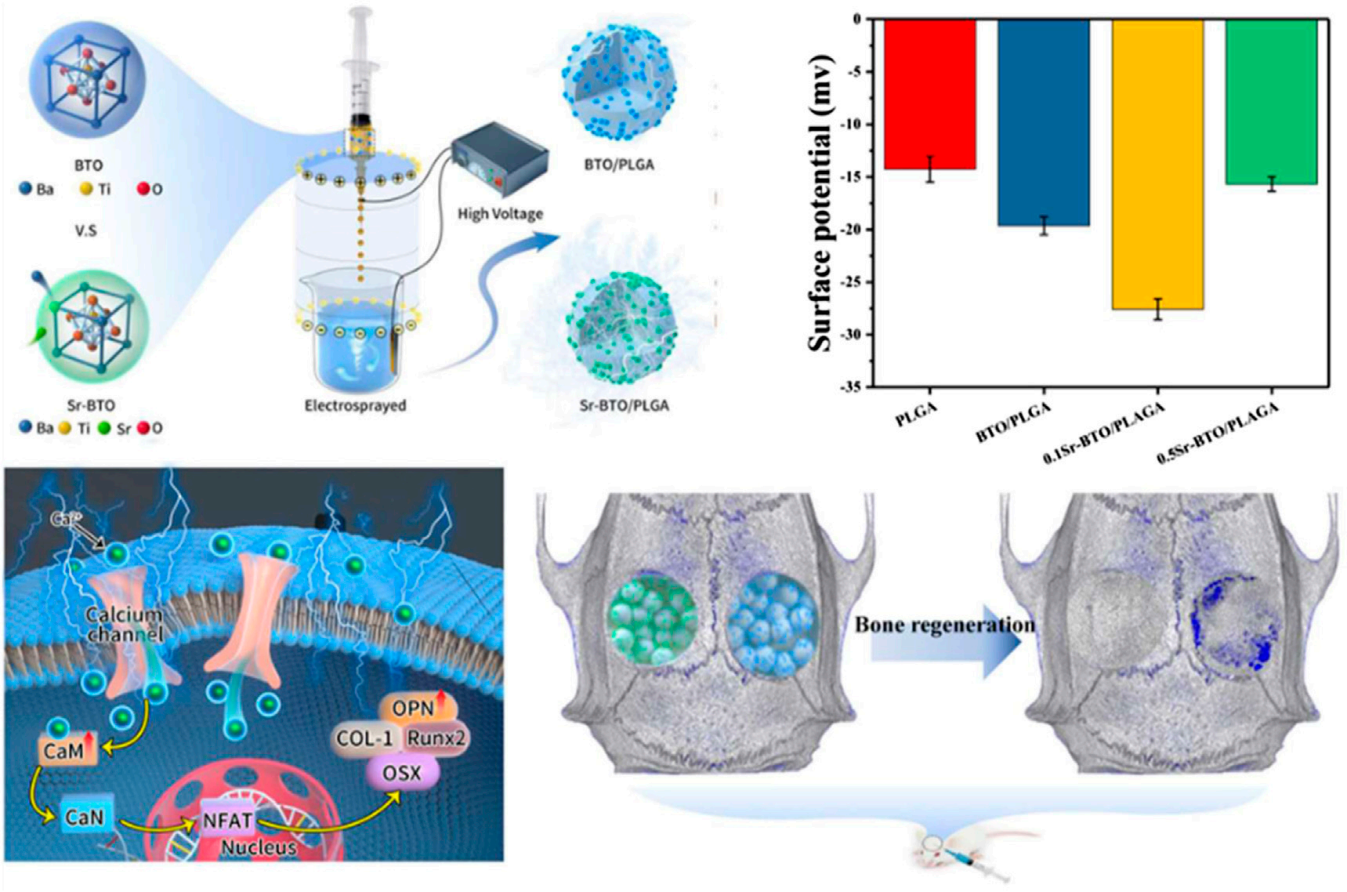
Schematic diagram of the fabrication process of Sr-BTO/PLGA microspheres and its application in bone repair.

With the ambition of imitating the bioelectrical properties of natural bones, as shown in Scheme 1 in this study, Sr-BTO NPs functionalized microspheres with different surface potential were fabricated by a high-voltage electrostatic (HEV). The microspheres with different electrical properties were modulated through regulating the phase composition of inorganic components in the microspheres by doping amount of Sr^2+^. *In vitro*, the osteogenic properties of microspheres with different surface potentials were compared and the potential mechanism of osteogenic differentiation regulated by Sr-BTO/PLGA microspheres was also evaluated and discussed. Finally, a calvarial defect model to further evaluate the potential of Sr-BTO/PLGA microspheres to induce bone regeneration *in vivo*.

## 2 Materials and methods

### 2.1 Materials

Barium hydroxide [Ba(OH)_2_·8H_2_O, 98%] was purchased from ACROS ORGANICS. Tetrabutyl titanate [Ti(OC_4_H_9_)_4_, 98%] was obtained from Energy Chemical. Strontium nitrate [Sr(NO_3_)_2_, 99%]. Ammonium hydroxide solution (28% in H2O, ≥ 99.99% mental basis) and acetic acid (ACS, 99.7%) was purchased from Aladdin. Ethanol (99.7%) was were acquired from XILONG SCIENTIFIC. PLGA (LA/GA = 80:20, Mn = 1x10^5^) was synthesized in our laboratory by the ring-opening copolymerization (ROP) of L-lactide (LA) and glycolide GA. Dulbecco’s modified Eagle’s medium (DMEM) was obtained from Gibco (New York, United States). Cell Counting Kit-8 (CCK-8) was purchased from Seven sea.

### 2.2 Hydrothermal synthesis of Sr-BTO NPs

A series of Ba_1-x_Sr_x_TiO_3_ powder were prepared by the hydrothermal method, using Ba(OH)_2_·8H_2_O, Ti(OC_4_H_9_)_4_, and Sr(NO_3_)_2_ as raw materials, where n(Ba): n(Ti) = 1.5:1. We labeled the Sr-BTO synthesized according to the ratio [n(Ba^2+^):n(Sr^2+^) = 7.9:0.1, 7.5:0.5] as 0.1Sr-BTO, 0.5Sr-BTO. Ethanol was added to tetrabutyl titanate with magnetic stirring. The amorphous TiO_2_ powder was precipitated by the additional 4 ml ammonium hydroxide and 5 ml deionized water to the solution mixture. Stoichiometric amounts of Ba (OH)_2_·8H_2_O and Sr (NO_3_)_2_ were dissolved in 10 ml deionized water in order in the proportions at 90°C. These precursors were mixed well and added to a hydrothermal reactor (the filling degree of the reactor was 60%). The reaction was carried out at 180°C for 240 h. After completion of the reaction the mixed solution was slowly cooled to room temperature. The reaction solution was adjusted to neutral with 0.05 mol/l acetic acid and the crude product washed twice with deionized water and hot ethanol, respectively, to obtain pure Sr-BTO nanoparticles.

### 2.3 Characterization of Sr-BTO NPs

The X-ray diffraction (XRD) was performed to confirm the crystal structure of Sr-BTO NPs using a D8 Advance diffractometer (Bruker Co., Germany). Fourier transform infrared (FTIR) spectroscopic analysis was carried out on a Perkin Elmer 580B IR spectrophotometer to analyze the chemical composition of Sr-BTO NPs. Further characterizing the phase composition of nanoparticles by Raman spectroscopy. The environmental scanning electron microscope (ESEM, XL30 FEG, Philips) was utilized to observe the morphologies and size of Sr-BTO NPs. The diameter of nanoparticles was measured by ImageJ according to SEM image. Determination the content of Ba^2+^ and Sr^2+^ in inorganic nanoparticles by Inductively coupled plasma atomic emission spectrometry (ICP-AES) (Thermo Jarrell Ash, USA). Furthermore, the ζ-potential of nanoparticles were analyzed by a zeta sizer (Malvern Zetasizer Nano Series).

### 2.4 Fabrication of Sr-BTO/PLGA microspheres

The Sr-BTO/PLGA microspheres was prepared by high-voltage electrostatic (HVE) technique as described in our previous method(Yan et al., 2021). Briefly, Sr-BTO NPs (40 wt% in Sr-BTO/PLGA) was dispersed in *N*-Methyl pyrrolidone solution (NMP) contained 8% PLGA (w/v) solution. Then the mixture solution was added to a 2.5-ml syringe and fixed it on the microsphere preparation device which is comprised of five parts: an extruder, an HVE generator (FRASER, UK), a stepper motor controller, a temperature controller, and a heating module. The nanocomposite microsphere were prepared under the high voltage electrostatic field of 6 kv. The solution flow rate was 2 ml/h and 60% ethanol solution as receiver solution. PLGA and BTO/PLGA microspheres were also prepared using the same preparation method.

### 2.5 Characterization of Sr-BTO/PLGA

The hydrophobicity of PLGA, BTO/PLGA and Sr-BTO/PLGA were assessed by water contact angle measurements performed on a video contact angle instrument (JC 2000C1, Shanghai Glory Numeral Technique and Device Co., Ltd., China). The inorganic component content of microspheres were characterized by thermogravimetric analysis (TGA) (TA Instruments TGA500, USA) with a heating rate of 10°C min^−1^ in N_2_ from 25 to 900°C. To evaluate the release behaviors of Sr^2+^ in 0.1Sr-BTO/PLGA and 0.5Sr-BTO/PLGA, 200 mg 0.1Sr-BTO/PLGA and 0.5Sr-BTO/PLGA microspheres were soaked in PBS (1 ml) at 37°C for 7, 14 and 21 days, respectively. Then 0.6 ml of microspheres extract at different point of time was taken out and analyzed by inductively coupled plasma optical emission spectrometry (ICP). The up and low surfaces of composites were coated with conductive silver paste as electrodes to test the dielectric properties at different frequencies from 10^2^ to 10^7^ Hz by broadband dielectric spectrometer (Novocontrol Concept80, Germany) at room temperature. The surface potential of microspheres was measured using an electrokinetic analyzer for solid sample (SurPASS 3, Anton Paar, Austria) in 0.1 M potassium chloride solutions at room temperature. The environmental scanning electron microscope (ESEM, XL30 FEG, Philips) was utilized to observe the morphologies of microsphere after being sputter-coated with platinum (30 mA, 90 s) using a sputter-coater (Polaron E5600, USA). EDS mapping was performed under the same parameters as SEM observation and recorded at 10 KV.

Dye adsorption properties: In order to evaluate the adsorption capacity of microspheres for charged small molecules, Sr-BTO/PLGA microspheres were evaluated over two representative dyes (Methylene blue (MB) and Methyl orange (MO) are typical cationic and anionic dyes). 10 mg microsphere was dispersed in 2 ml dye solution (5 mg/l) and then the suspension was stirred for 1 h in the dark to reach adsorption desorption equilibrium between the dye and microspheres. The absorbance values of methyl orange and methyl blue after adsorption were measured at 463 and 662 nm using a multi-function microplate reader, respectively.

### 2.6 Cell culture

Mouse embryo osteoblast precursor cells (MC3T3-E1) cells (Shanghai Institutes for Biological Sciences, Chinese Academy of Sciences) were selected for *in vitro* biological evaluation and cultured in Dulbecco’s Minimum Essential Medium (Gibco, New York, United States) with FBS (10%, Gibco), 100 mg l^−1^ streptomycin (Sigma), and 63 mg l^−1^ penicillin (Sigma). The details of the cellular assay are available in the Supporting Information.

### 2.7 *In vivo* osteogenic capacity of Sr-BTO/PLGA microsphere

All animal experiments implemented in this study were approved by the ethical committee of Changchun Institute of Applied Chemistry Chinese Academy of Sciences, and the experiments were consistent with the Guide for the Care and Use of Laboratory Animals. Sprague-Dawley SD rat (female, 12 weeks, body weight: 250-300g) were purchased from Liaoning Changsheng Biotechnology Co., Ltd. (Liaoning, China). Critical-size cranial defect model (5 mm) were applied to evaluate the bone regeneration effect of microspheres *in vivo*. The detailed surgical operation was described in supporting information. At 4 and 8 weeks after surgery, animals were sacrificed by intraperitoneal injection of pentobarbital. The rat skulls were collected and then fixed with 4% PFA for 48 h. The regeneration of calvarial defects was evaluated by assessing the morphology of skulls utilizing a micro computed tomography (micro-CT) scanner (SkyScan 1,172, Bruker). Besides, *in vivo* T_1_-weghted images were recorded on a 1.2 T Magnetic Resonance Imaging (MRI) Scanner (HT-MRSI50-50KY, Shanghai, China). After Micro-CT and MRI analysis, calvarial bone from each group were then used for histological observation. The calvarium samples were decalcified in 10% EDTA solution (pH 7.0) for 28 days, dehydrated in a graded series of ethanol and embedded in paraffin for sectioning. Subsequently, the hematoxylin-eosin (H and E), Masson trichrome, and picrosirius red staining were used for visualization of the new bone formation and collagen deposition. Capture the staining images by optical microscopy.

### 2.8 Statistical analysis

Measurements were performed in triplicate if not otherwise stated. The obtained data are expressed as mean ± standard deviation values. Statistical analysis was determined by one-way analysis of variance (ANOVA) with Origin software. The *p* < 0.05 values labeled with an asterisk (*) denotes significant differences.

## 3 Results and discussion

Endogenous electric fields generated by bone play an important role in the process of bone repair and regeneration (Oconnor et al., 1969). In view of this, simulating the electrical properties of bone and constructing a surface potential microenvironment may be an effective means to accelerate osteogenic differentiation and enhance bone regeneration. The 0-3 type nanocomposites as functional materials can acquire properties that cannot be obtained in single polyester materials by adding functional inorganic particles (0 represents functional nanoparticles and three represents polymer matrix phase). Therefore, in this study, composite microspheres were prepared by introducing electroactive nanoparticles into PLGA matrix with high voltage electrostatic to restore the electrical microenvironment.

### 3.1 Characterization of Sr-BTO nanoparticles

#### 3.1.1 The effect of Sr doping on physicochemical characterization of BTO NPs

**FIGURE 1:**
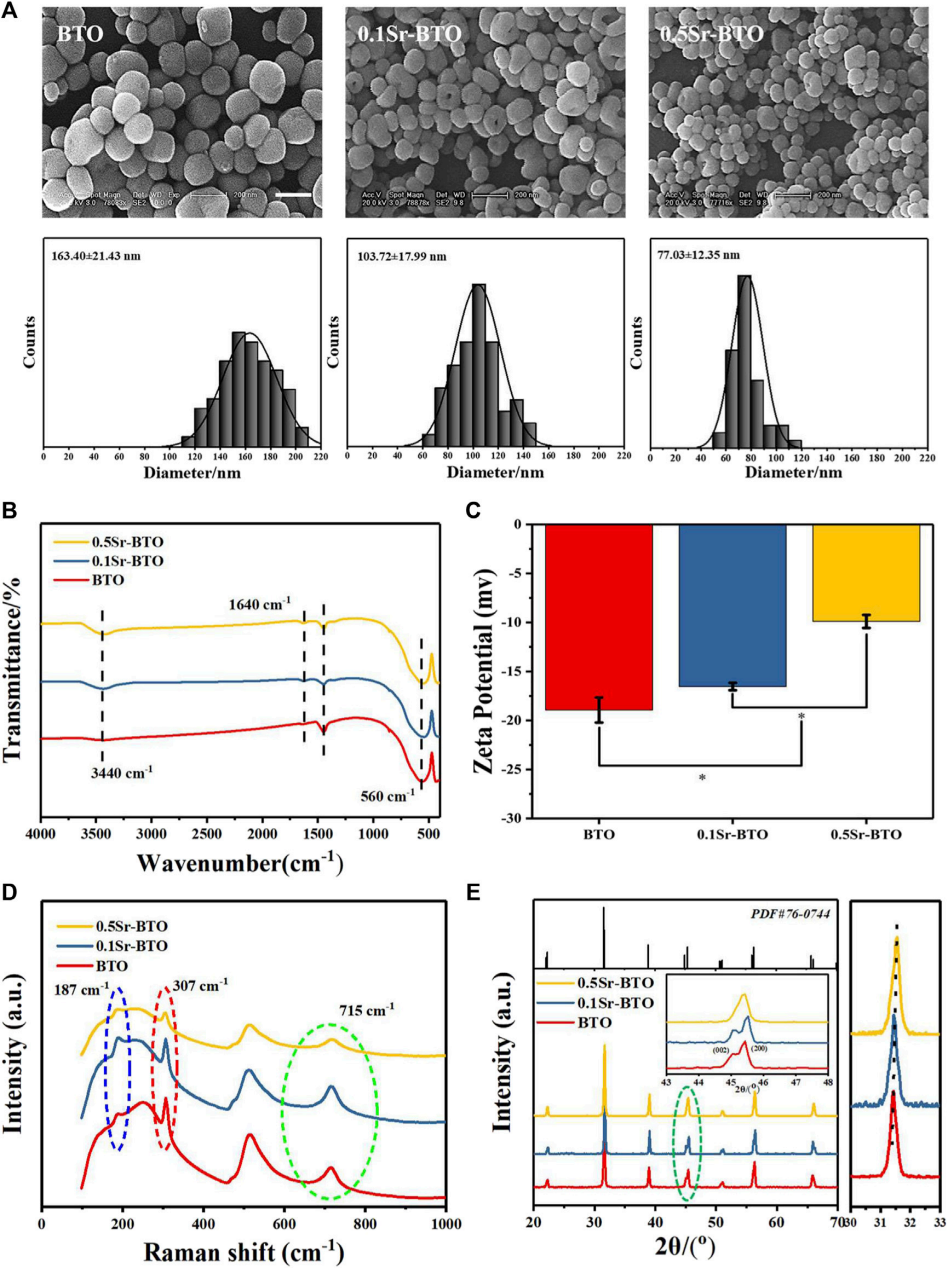
**(A)** SEM images and diameter distribution histograms of BTO and Sr-BTO nanoparticles. Scale bar: 200 nm. **(B)** FT-IR spectra **(C)** Zeta potential **(D)** Raman spectra, **(E)** XRD patterns (E, left), the XRD step scan from 30 to 33 (E, right) of BTO and Sr-BTO NPs.

Firstly, Sr-BTO electroactive nanoparticles were obtained by hydrothermal method. The SEM images of nanoparticles were displayed in Figure 1A, like BTO NPs, the shapes of 0.1Sr-BTO NPs and 0.5Sr-BTO NPs were presented a ball shape which shows that the introduction of Sr^2+^ has almost no effect on the morphology of nanoparticles. However, the introduction of Sr^2+^ could reduce the particle size. The average diameters of BTO, 0.1Sr-BTO and 0.5Sr-BTO were 163.40 ± 21.43, 103.72 ± 17.99, and 77.03 ± 12.35 nm, respectively, via Image J. The particle size of nanoparticles decreases with the increase of Sr^2+^ content. To further explore the effect of Sr^2+^ on the chemical component of barium titanate, FT-IR was performed and depicted in Figure 1B. From the FT-IR spectrum, the absorption peaks at 560 cm^−1^ were attributed to the titanium oxide octahedral structure of BTO and Sr-BTO NPs. The peaks at 3,440 and 1,640 cm^−1^ arose from the O-H telescopic vibration and bending vibration, respectively, indicating that the sample contained adsorbed water. Furthermore, the molar ratio of Ba^2+^ to Sr^2+^ in nanocrystals was determined by ICP. According to Supplementary Table S1, the chemical formulas of 0.1Sr-BTO and 0.5Sr-BTO were determined as Ba_0.98_Sr_0.02_TiO_3_ and Ba_0.93_Sr_0.07_TiO_3_, respectively, which were close to the results calculated by feeding ratio. The zeta potential depends on the chemical composition, surface properties, and particle size of nanoparticles (El-Ghannam et al., 2001). The zeta potentials of nanoparticles at pH 7.4 were -18.93 ± 1.27, -16.53 ± 0.38, -9.89 ± 0.66 mV for BTO, 0.1Sr-BTO and 0.5Sr-BTO NPs respectively (Figure 1C). According to the FTIR, the Sr-BTO microstructure contains OH^−^, which causes the nanoparticles to exhibit a negative potential. With the increase of Sr^2+^ incorporation, there is lattice distortion caused by ionic radius difference which in turn alternates the surface charge, resulting in an increase in the zeta potential, which similar to the result of Sr^2+^ doped mesoporous bioactive glass (Amudha et al., 2020). These above results indicated that electroactive nanoparticles with different Sr contents (0.1Sr-BTO NPs and 0.5Sr-BTO NPs) were obtained by hydrothermal method.

#### 3.1.2 The effect of Sr doping on phase composition of BTO NPs

The electrical properties of BTO are closely related to its phase composition. Among the different crystals of perovskite BTO series, tetragonal phase barium titanate has excellent electrical properties (dielectric, ferroelectric and piezoelectric). To explore the influence of Sr^2+^ doping on the crystal structure of nanoparticles, we performed Raman and XRD on nanoparticles. The cubic phase BTO belongs to the Pm3m point group, the tetragonal phase BTO unit cell space group belongs to P4mm, and generally considered that the Pm3m point group has no Raman activity. To evaluate the effect of Sr^2+^ on the crystal phase of BTO, Raman shift spectra of BTO, 0.1Sr-BTO and 0.5Sr-BTO NPs are shown in Figure 1D. The peaks around 715, 515, 307, 260, and 185 cm^−1^ are attributed to the characteristic peaks of BTO, wherein 185, 715 and 307 cm^−1^ attributed to characteristic peaks of the tetragonal phase (Pasha et al., 2007). Therefore, BTO and Sr-BTO NPs still possesses a tetragonal phase structure. However, for 0.5Sr-BTO NPs, the peaks at 715 and 307 cm^−1^ are significantly reduced, indicating that the content of tetragonal crystals in 0.5Sr-BTO is significantly reduced. The XRD patterns of BTO and Sr-BTO NPs were obtained to further explore the effect of Sr^2+^ on the crystal composition of nanoparticles (Figure 1E). Compared to the standard data (PDF No. 76-0,744) of tetragonal BTO, the diffraction characteristic peaks of at 22.2, 31.5, 38.9, 44.9, 45.3, 50.7, 56.3, and 65.7 correspond to the (100), (101), (111), (002), (200), (102), (211), and (202) crystal planes in the pure tetragonal crystals, respectively. These results showed that no obvious second phase is found in 0.1Sr-BTO NPs and 0.5Sr-BTO NPs, which indicated that introduction of Sr^2+^ did not lead to the formation of other impurity phases. Moreover, the diffraction peaks (101) in the XRD patterns (Figure 1E, right) shift toward a higher degree, which may be due to the shrinkage of the unit cell caused by the substitution of Sr^2+^ with a smaller radius for Ba^2+^ with a larger radius. It is worth noting that at around 45 degrees, the diffraction peaks of (002) and (200) planes can be clearly observed in BTO and 0.1Sr-BTO NPs. However, in the 0.5Sr-BTO diffraction peak, only the (200) crystal plane can be observed around 45 degrees. These results indicated that the 0.5Sr-BTO NPs are transformed from the original tetragonal phase to the cubic phase, which is consistent with the Raman results. The decrease of tetragonal phase content in 0.5 Sr-BTO will hinder its electrical properties. In addition, the Δ2θ of the (002) and (200) planes (Δ2θ = 2θ_(200)_- 2θ_(002)_) was calculated and found that the Δ2θ of 0.1Sr-BTO NPs (0.430) was higher than that of BTO NPs (0.382), which indicated that doping of a small amount of Sr^2+^ contributes to the enhancement of the tetragonal phase content in the crystal, and the electrical performance is speculated significantly enhanced. Therefore, the use of 0.1Sr-BTO NPs as the inorganic component in the composite is expected to endow the material with higher electrical properties.

#### 3.1.3 Sr-BTO NPs toxicity evaluation

BTO NPs are considered as nanomaterials with extensive biomedical value (Dai et al., 2021), but the biological toxicity of Sr-BTO NPs still needs further evaluation. To verify whether Sr-BTO NPs could be used in biological studies, it is necessary to evaluate the cytotoxicity of the nanoparticles. Referring to the previous method of evaluating the toxicity of nanoparticle, cells were cultured with nanoparticles leaching liquors and their 1/2, 1/4 and 1/8 diluents, respectively (Hao et al., 2019). The cells were cultured with high-glucose Dulbecco’s modified Eagle’s medium as control group. As shown in Supplementary Figure S1C, the cell viability was evaluated via CCK-8 assays indicated that the viabilities of cells treated with different leaching liquors were not less than 80%. Apart from this, the morphology of MC3T3-E1 cells treated with medium suspensions (62.5 μg/ml) of BTO and Sr-BTO NPs and their extracts was normal (Supplementary Figures S1A,B), exhibiting a typically fusiform cell shape and spreading well. These results indicated that BTO and Sr-BTO NPs have no obvious toxicity to cells, and it is safe to apply to tissue repair materials.

### 3.2 Preparation of Sr-BTO/PLGA microsphere

#### 3.2.1 General physicochemical properties

**FIGURE 2:**
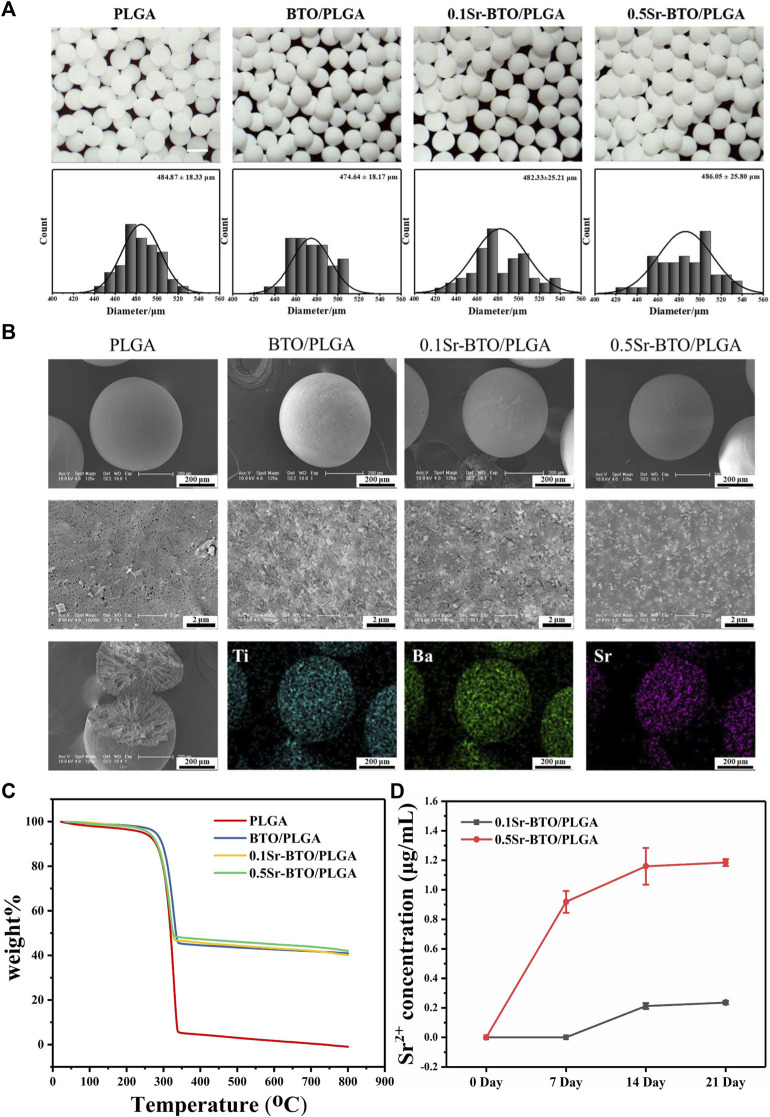
**(A)** The micrographs and statistical analysis of the size distribution of microsphere. Scale bar: 500 μm **(B)** The SEM image and Mapping image of elemental compositions (including Ti, Ba and Sr elements distribution of 0.1Sr-BTO/PLGA) of microspheres. **(C)** TGA analysis of Sr-BTO/PLGA microspheres. **(D)** Sr^2+^ release concentration of Sr-BTO/PLGA microspheres immersed in PBS at 0, 7, 14 and 21 days.

The Sr-BTO/PLGA microspheres were prepared by an high-voltage electrostatic (HVE) technique, which is based on the balance between electrostatic force and solution surface tension (Li et al., 2019). In addition, the preparation method assisted by the high-voltage electrostatic field could contribute to the polarization of the electroactive nanoparticles to improve the electrical properties of composite (Li et al., 2017). The gross morphology and particle size distribution of the microspheres were observed by stereo optical microscope. As shown in Figure 2A, we obtained uniform microspheres containing different nanoparticles through screening, displaying an average diameter of ∼ 480 μm. These microspheres exhibited excellent sphericity and homogeneity, and are expected to be used as scaffold biomaterials for osteoblasts. It can be observed from the SEM image that the nanoparticles were more evenly scattered on the surface of the microspheres at the nanoscale (Figure 2B). The cross-sectional image shows that the microspheres have a porous interior structure that facilitates the delivery of nutrients and tissue invasion. Furthermore, the chemical elemental compositions of the microspheres were determined using EDS mapping analysis. The representative images of element mapping were shown in Figure 2B, Ti, Ba and Sr elements were clearly observed on the 0.1Sr-BTO/PLGA microsphere surface and distributed homogenously in the microspheres. Therefore, microspheres with uniformly distributed electroactive nanoparticles could be prepared under the assistance of high-voltage electrostatic fields.

To determine the actual content of nanoparticles in the microspheres, the weight content of Sr-BTO NPs in Sr-BTO/PLGA microspheres were evaluated by TGA analysis. As shown in Figure 2C, the weight loss at temperatures below 200°C was attributed to the evaporation of moisture. The residual weight above 350°C resulted mainly from nanoparticles. The contents of inorganic components in the BTO/PLGA, 0.1Sr-BTO/PLGA and 0.5Sr-BTO/PLGA microspheres were 40.92%, 40.19% and 41.99%, respectively, which corresponded closely to the theoretical values (40%). There was no significant difference in the content of inorganic components among BTO/PLGA, 0.1Sr-BTO/PLGA, and 0.5Sr-BTO/PLGA microspheres, which suggested that the differences in the physicochemical properties and the subsequent biological activity of the microspheres in this study has little correlation with the content of inorganic components. In addition, as shown in Supplementary Figure S2, the contact angles of PLGA, BTO/PLGA, 0.1Sr-BTO/PLGA and 0.5Sr-BTO/PLGA demonstrated that the introduction of nanoparticles does not affect the hydrophilicity of the composite (*p* > 0.05). Furthermore, Sr^2+^ is widely believed to promote osteogenic differentiation and play a positive role in bone regeneration (Zhang et al., 2018). It is necessary to evaluate the release behaviors of Sr^2+^ in the Sr-BTO/PLGA microspheres. As shown in Figure 2D, the concentration of Sr^2+^ released from the 0.1Sr-BTO/PLGA microspheres was significantly lower than that released from the 0.5Sr-BTO/PLGA microspheres at all time points. Interestingly, after the microspheres were soaked in PBS for 7 days, the presence of Sr^2+^ was not detected in the 0.1Sr-BTO/PLGA group. When the microspheres were soaked in PBS for 14 and 21 days, the Sr^2+^ concentration in the solution of 0.1Sr-BTO/PLGA and 0.5Sr-BTO/PLGA remained almost unchanged, which indicated that the Sr^2+^ were released almost completely after the microspheres were soaked in PBS for 14 days. The final release concentrations of strontium ions from 0.1Sr-BTO/PLGA and 0.5Sr-BTO/PLGA microspheres was only 0.23 ± 0.01 and 1.18 ± 0.02 μg/ml, which was lower than the effective concentration range (2-6 μg/ml) for promoting osteogenic differentiation in related reports (Naruphontjirakul et al., 2018). These above results suggested that Sr^2+^ released from Sr-BTO/PLGA microspheres have limited impact on subsequent biological evaluation.

#### 3.2.2 Electrical performance

**FIGURE 3:**
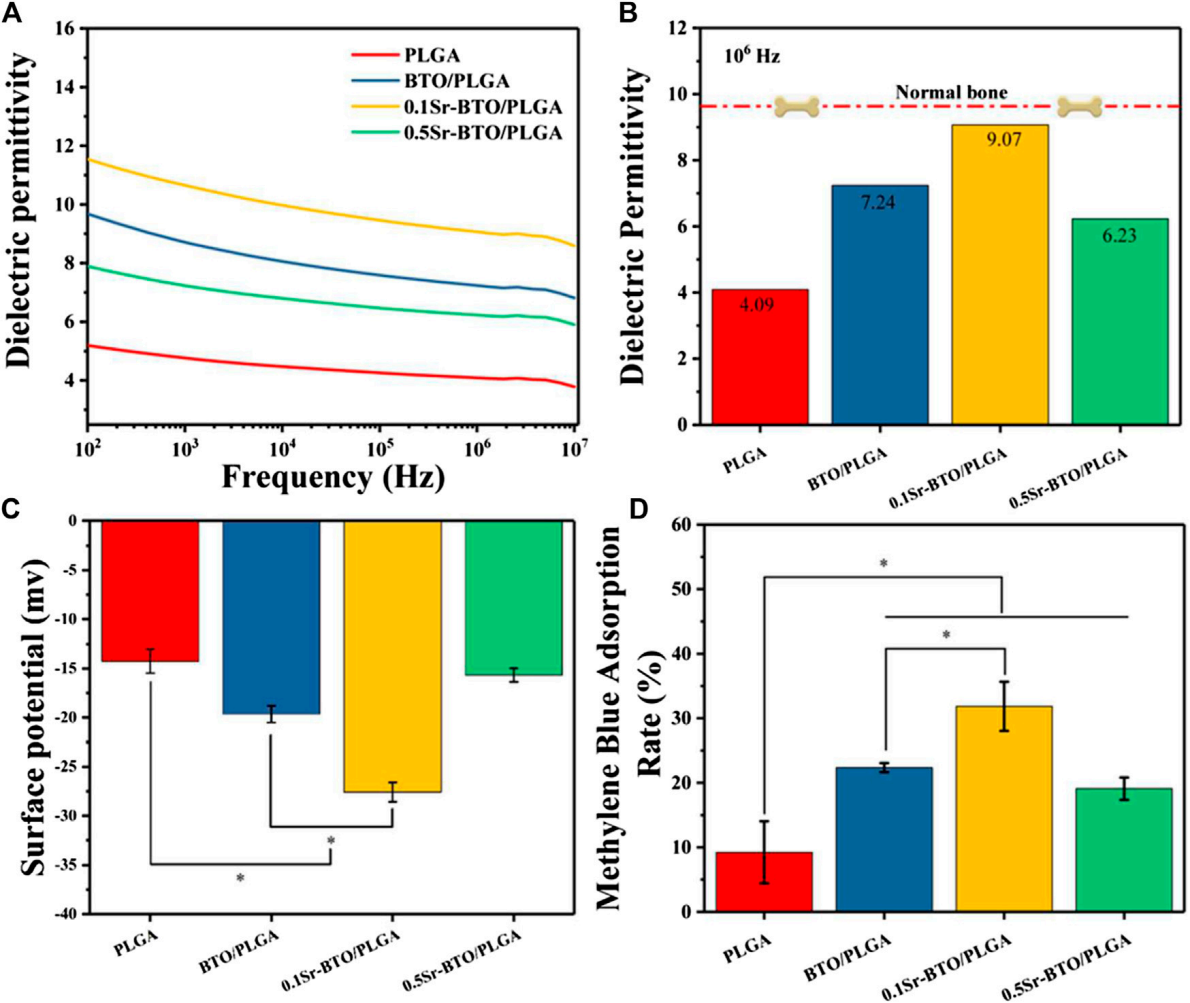
**(A)** Dielectric permittivity of Sr-BTO/PLGA. **(B)** Dielectric permittivity at 10^6^ Hz of the Sr-BTO/PLGA was compared with that of normal bone (red line) **(C)** Zeta potential of Sr-BTO/PLGA microspheres at room temperature. **(D)** Adsorption rate of Sr-BTO/PLGA microspheres for methylene (MB) blue dyes.

Physiological potentials are widely present in cells and organs, among which the electrical properties of bone have attracted widespread attention. In natural living bone, the hydrogen bond in collagen and hydroxyapatite is responsible for its polarizability. The dielectric behavior of bone depends on the frequency and moisture content. In this work, we attempt to modulate the electrical properties of composites by introducing electroactive nanoparticles to simulate electrical properties of nature bone. In order to study the influence of the introduction of electroactive nanoparticles on the electrical properties of composites, we studied the dielectric properties of the composites. As shown in Figure 3A, the frequency-dependent dielectric permittivity results at 10^2^ ∼ 10^7^ Hz of PLGA and Sr-BTO/PLGA composites. The introduction of electroactive nanoparticles could improve the dielectric properties of the composite materials. The dielectric permittivity at 10^6^ Hz of PLGA, BTO/PLGA, 0.1Sr-BTO/PLGA and 0.5Sr-BTO/PLGA were 4.09, 7.24, 9.07, and 6.23, respectively, wherein 0.1Sr-BTO/PLGA were much closer to the measured value of normal bone (∼9.6) reported by Amin et al. (Amin et al., 2019). According to the result of Raman and XRD spectrum, the characteristic peak of the tetragonal phase intensity of 0.5Sr-BTO is obviously reduced, which was the main reason that the electrical activity of the 0.5Sr-BTO/PLGA group is weaker than that of the BTO/PLGA and 0.1Sr-BTO/PLGA groups. The electrical activity of the 0.1Sr-BTO/PLGA group is higher than that of the BTO/PLGA group, which may be since the higher content of tetragonal phase in 0.1Sr-BTO. Furthermore, the interface area is also the key factor affecting the dielectric properties of the 0-3 type composites. Under the action of the electric field, the charges are easy to accumulate at the interface, and the local accumulation of space charges leads to uneven distribution of charges in the dielectric, resulting in macroscopic electric moments and interface polarization. Under the same inorganic content, the smaller the particle size of nanoparticles, the larger the interface area between nanoparticles and matrix, which lead to form the greater the interface polarization. In this study, PLGA matrix was filled with smaller nanoparticle size 0.1Sr-BTO NPs could increase the interface area in the composite, strengthen the interface bonding, and finally effectively improve the dielectric properties of the composite. From this point of view, the dielectric properties of 0.1Sr-BTO/PLGA is closer to that of bone, which is expected to have great application potential in bone regeneration.

Physiological potential microenvironment plays an indispensable role in regulating cell differentiation and maintaining bone volume and quality (Zhang et al., 2016). It is necessary to measure the surface potential of the microspheres by solid surface ζ-potential tester. The surface potential of the microsphere is based on the measurement of the streaming potential, which arises from the flow of a liquid across charged surfaces. As shown in Figure 3C, the surface potential for PLGA, BTO/PLGA, 0.1Sr-BTO/PLGA, and 0.5Sr-BTO/PLGA microsphere are -14.26 ± 1.21, -19.64 ± 0.85, -27.58 ± 0.98, -15.67 ± 0.69 mV, respectively. These results indicated that the introduction of nanoparticles leads to a decrease in the surface potential on microspheres, wherein the surface potential of the BTO/PLGA and 0.1Sr-BTO/PLGA microspheres changes more obviously, which was related to the tetragonal phase content in nanoparticles. The surface potential of 0.5Sr-BTO/PLGA microspheres is slightly lower than that of PLGA, but there is no statistical difference. Furthermore, compared with BTO/PLGA microsphere, the 0.1Sr-BTO/PLGA microsphere possessed a lower surface potential. It is speculated that 0.1Sr-BTO/PLGA has larger dielectric constants, which is more conducive to the storage of charges during the preparation of microspheres by high-voltage electrostatics. Therefore, the surface potential of microspheres can be effectively modulated by adjusting the doping amount of Sr^2+^.

Moreover, there are a lot of charged substances in the physiological environment, including solution ions, proteins and so on. When biomaterials are implanted into organisms, different charged surfaces may adsorb different charged small molecules that affect cell adhesion, proliferation, and differentiation. To further confirm the adsorption of surface potential, anionic (Methyl Orange, MO) and cationic (Methylene blue, MB) dyes were employed as models of charged small molecules in a physiological environment to evaluate the adsorption capacity of the microspheres for charged species (Dai et al., 2019). The type of surface charge is widely considered as the key factor that influence the adsorption behavior of adsorbent (Kang et al., 2020). Organic dyes usually have affinity towards materials with opposite-charge. Compared with the anionic dye MO (Supplementary Figure S3), the adsorption capacity of all microspheres to the cationic dye MB (Figure 3D) is stronger, which due to the negative potential microenvironment on the surface of microspheres. Furthermore, the result of MB adsorption showed that the cation adsorption ability of the microspheres was significantly improved after adding electroactive nanoparticles, which is because the introduction of electroactive nanoparticles can reduce the surface potential on the microspheres. Especially, the 0.1Sr-BTO/PLGA microspheres had the strongest ability to cationic dyes, which is due to the lowest surface potential. The enhanced adsorption capacity of microspheres for cations is expected to facilitate the adsorption of positively charged ions (Ca^2+^, Na^+^, etc) to regulate the behavior of cells in physiological environments. In summary, the 0.1Sr-BTO/PLGA microspheres have lower surface potential and bone-matched dielectric properties, which are expected to have great potential in regulating cell behavior and promoting bone tissue regeneration.

### 3.3 Biocompatibility of Sr-BTO/PLGA microspheres *in vitro*

#### 3.3.1 Cellular spreading and proliferation

**FIGURE 4:**
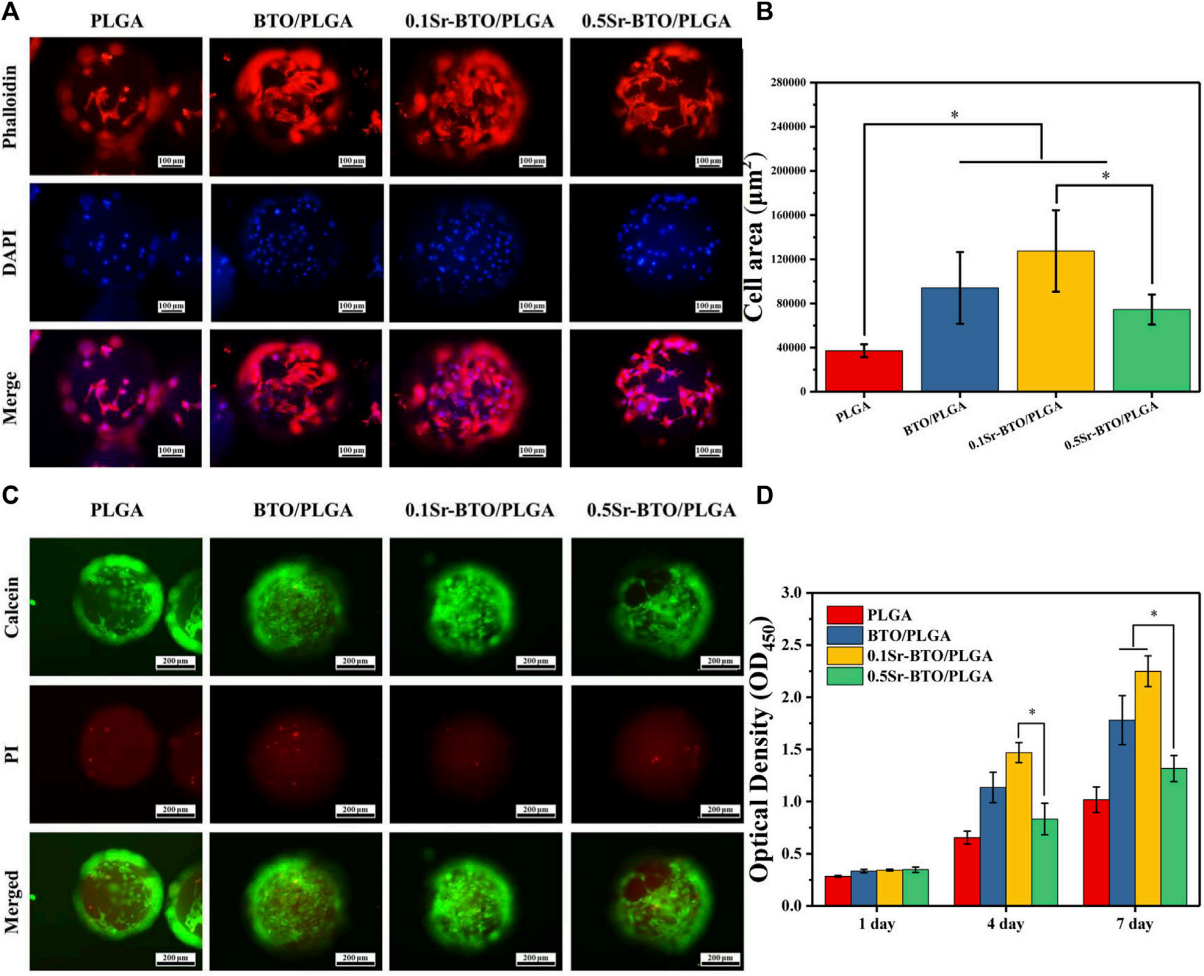
**(A)** Fluorescence micrographs and **(B)** the corresponding cell area per field of MC3T3-E1 cells cultured on PLGA, BTO/PLGA, Sr-BTO/PLGA microspheres for 3 days. Cell was stained with phalloidin (red) and DAPI (blue). Scale bar: 100 μm. **(C)** Representative fluorescence images showing live calcein-stained (green) and dead PI-labeled (red) MC3T3-E1 after culturing in microspheres for 7 days. Scale bar: 200 μm. **(D)** CCK-8 assay of MC3T3-E1 cultured on different microsphere at different culture time. *p < 0.05.

Bioactive materials allow cell adhesion spreading, and tissue crawling, which played a very necessary role in bone regeneration. Hence, the attachment behavior of MC3T3-E1 cells cultured on different microspheres were evaluated by fluorescence staining (4′,6-diamidino-2-phenylindole (DAPI) and Phalloidin). The representative images and quantitative results of cell spreading area are shown in Figures 4A,B, in which blue represents the nucleus and red represents the actin cytoskeleton. As revealed in the fluorescence staining and quantitative results, the microspheres containing electroactive nanoparticles could ameliorate the adhesion behavior of cells in comparison with that of PLGA microspheres, which may be due to decrease the surface potential on the microspheres by introduction of nanoparticles. In addition, 0.1Sr-BTO/PLGA microspheres were more conducive to cell spreading and adhesion. More importantly, compared with 0.5Sr-BTO/PLGA microsphere group, the cells on the surface of 0.1Sr-BTO/PLGA microspheres had a better spreading form. Therefore, we speculated that the lower surface potential of the microspheres is more conducive to promoting cell adhesion. It is conjectured that the surface potential generated on microspheres may induce the readjustment of the protein structure located on the cell membrane surface, such as fibronectin, and affect cell adhesion and spreading (Tang et al., 2018). Another possible reason is that the low negative surface potential favors the adsorption of positively charged ions or molecules (Figure 3D). Cell membranes are negatively charged, which are more conducive to adhesion to the surfaces enriched in positively charged ions and molecules (Marchesano et al., 2015).

Live/dead staining was performed on cell/microsphere complexes by staining with calcein-AM/propidium iodide (PI) after 7 days of culture. As revealed in Figure 4C, living cells adhering and spreading on all groups of microspheres were dominantly stained green while dead cells (red fluorescence) were almost invisible, which indicated strong cell viability. Therefore, BTO/PLGA and Sr-BTO/PLGA microspheres were also identified noncytotoxic and had excellent biocompatibility, which could be further used in differentiation studies. Furthermore, the cell viability was measured by CCK-8. Figure 4D shows the proliferation results of MC3T3-E1 cells cultured on microspheres for 1–7 days. On the first day, the optical density value (OD values) of BTO/PLGA, 0.1Sr-BTO/PLGA and 0.5Sr-BTO/PLGA groups were slightly higher than that of PLGA microspheres, which demonstrated that microspheres containing electroactive nanoparticles are more conducive to cell adhesion. Moreover, continuous cell growth was detected in all microspheres from 1 to 7 days, wherein the cells cultured on 0.1Sr-BTO/PLGA microspheres maintained the better proliferation state at all time points. Interestingly, at 4th day, 0.1Sr-BTO/PLGA microspheres can better promote cell proliferation in comparison with 0.5Sr-BTO/PLGA group. Furthermore, after the cells were cultured on the surface of the microspheres for 7 days, the cell viability of the BTO/PLGA and 0.1Sr-BTO/PLGA microsphere groups was significantly higher than that of the 0.5Sr-BTOPLGA group. These above results suggested that the electroactive nanoparticle-functionalized microspheres could better promote cell proliferation and lower surface potential is more conducive to promoting cell proliferation and adhesion. Taken together, the 0.1Sr-BTO/PLGA microspheres had the ability to maintain cell viability and promote cell spreading.

#### 3.3.2 Osteogenic differentiation

**FIGURE 5:**
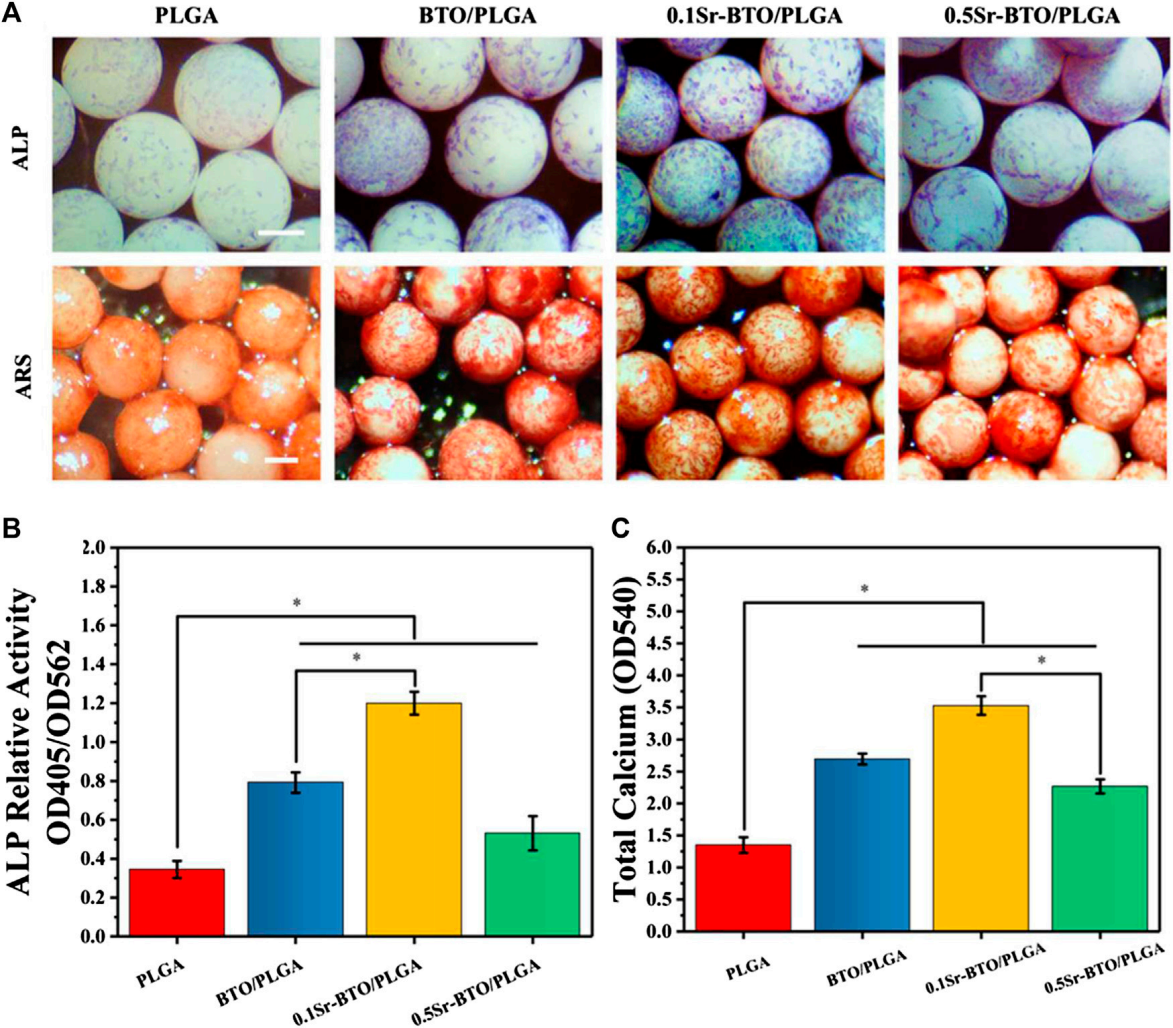
**(A)** The ALP and the Alizarin Red staining of MC3T3-E1 cells cultured on microspheres. Scale bar: 200 μm **(B)** The quantitative evaluation of ALP relative activity of MC3T3-E1 cells cultured on microspheres for 7 days **(C)** The Alizarin Red staining quantitative evaluation of calcium content mineral deposition in MC3T3-E1 cells cultured on microspheres for 14 days, *p < 0.05.

In addition to facilitating cell adhesion and spreading, the desirable bone repair material should have the potential to promote osteogenic differentiation. The osteogenic differentiation of cells on microspheres was evaluated in terms of alkaline phosphatase staining (ALP), alizarin red staining (ARS), and extracellular matrix element analysis. ALP is a byproduct of osteoblasts and the increase in ALP activity is closely related to bone formation. As shown in Figure 5A, the cells cultured on BTO/PLGA, 0.1Sr-BTO/PLGA and 0.5Sr-BTO/PLAG microspheres showed positive staining results on 7 days in comparison with PLGA microspheres. Among them, 0.1Sr-BTO/PLGA microspheres group exhibited the highest ALP activity due to the lower surface potential combined with ALP quantitative results (Figure 5B). Furthermore, compared with 0.5Sr-BTO/PLGA microsphere, the BTO/PLGA microsphere group showed more favorable promotion of ALP expression, which suggested surface potential is the main factor affecting ALP expression. ARS is suitable for the staining of calcium deposition and widely used in the study of ECM mineralization. The result of ARS staining showed a similar trend as the results of ALP activity that abundant mineralization nodules were induced on microspheres contained nanoparticles. Besides, the quantitative relative values measured at 405 nm by a microplate reader were also in accordance with the staining results (Figure 5C). It is worth noting that a considerable number of calcium nodules were observed on the 0.1Sr-BTO/PLGA microspheres while the osteogenic differentiation ability of 0.5Sr-BTO/PLGA microspheres was weaker than that of 0.1Sr-BTO/PLGA and BTO/PLGA microspheres. In order to further confirm the effect of chemical composition of chemical materials on osteogenic differentiation, ALP and ARS staining and quantitative analysis were also performed on the cells cultured on the composite membrane. Remarkably, these results showed that the membranes containing electroactive nanoparticles did not promote the increase in ALP activity and the formation of calcium nodules (Supplementary Figure S4). Based on the ALP and Alizarin Red staining results of the microspheres and composite, we speculate that the main reason for regulating the osteogenic differentiation by microsphere can be attributed to the surface potential of the microspheres. In other words, the microspheres contained nanoparticles prepared by the high-voltage electrostatic method may be more beneficial to improve the electrical activity under the polarization of the high-voltage electrostatic field, which are conductive to accelerating osteogenic differentiation.

**FIGURE 6:**
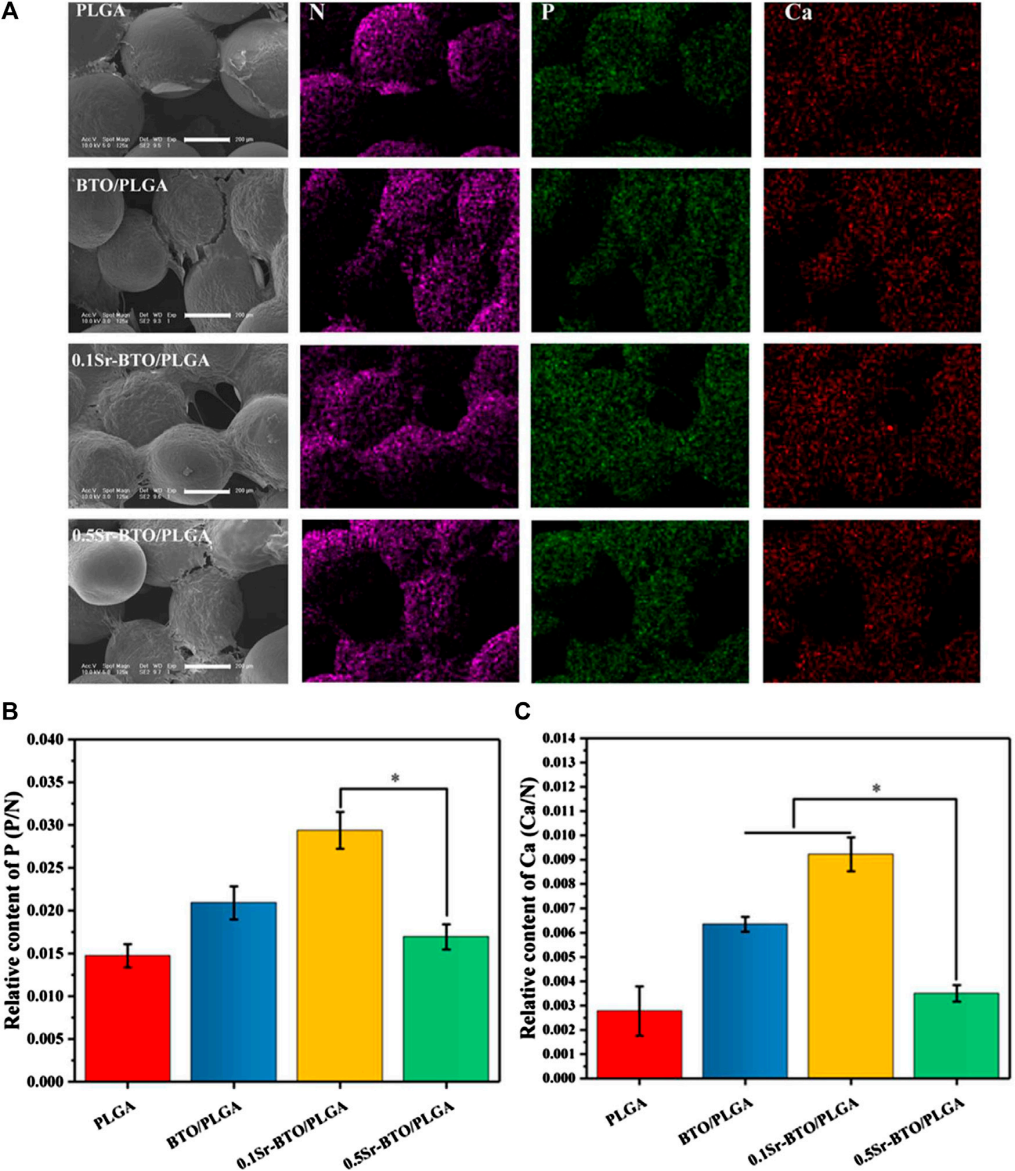
**(A)** The SEM and mapping image of MC3T3-E1 cells cultured on composite microspheres for 14 days. Scale bar: 200 μm **(B,C)** The relative contents of phosphorus (P) and calcium (Ca) in the extracellular matrix on different microsphere groups were calculated by N normalization, *p < 0.05.

Furthermore, SEM and EDS mapping were further used to observe element distribution and detected the mineralization extracellular matrix (ECM) in different microspheres, after 14 days of cell cultured on microsphere. As shown in the SEM image (Figure 6A), the secretion amounts of ECM in BTO/PLGA and Sr-BTO/PLGA microspheres were greater than those in PLGA microspheres. From the EDS mapping image, more calcium and phosphorus were observed on the ECM of cells cultured on BTO/PLGA and Sr-BTO/PLGA microspheres, which indicated that electroactive nanoparticles introduced into the microspheres contribute to promoting ECM mineralization. Furthermore, according to EDS, the relative contents of phosphorus (P) and calcium (Ca) in the extracellular matrix on different microsphere groups were calculated by N normalization (Figures 6B,C). Interestingly, compared to 0.5Sr-BTO/PLGA microspheres, the cells cultured on 0.1Sr-BTO/PLGA microspheres could mineralize more Ca and P for 14 days. Similar to ALP and ARS results, 0.5Sr-BTO/PLGA group did not significantly promote osteogenic differentiation compared with BTO/PLGA and 0.1Sr-BTO/PLGA group. Therefore, in this study, these results implied that the surface potential of the microspheres play a more important role in osteogenic differentiation than the release of Sr^2+^.

**FIGURE 7:**
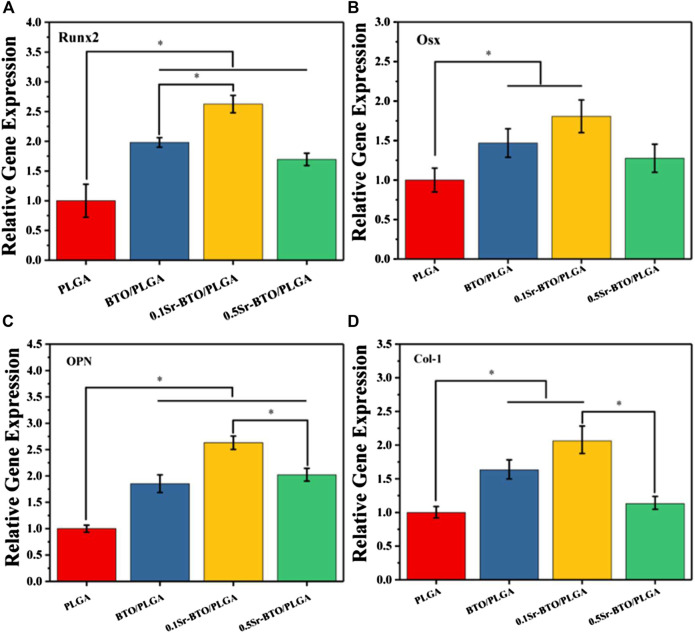
RT-PCR results of MC3T3-E1 cultured on different microspheres for 7 days: relative gene expression levels of **(A)** Runx 2, **(B)** Osx, **(C)** OPN, and **(D)** Col-1; GADPH was used as a reference gene. *p < 0.05.

**FIGURE 8:**
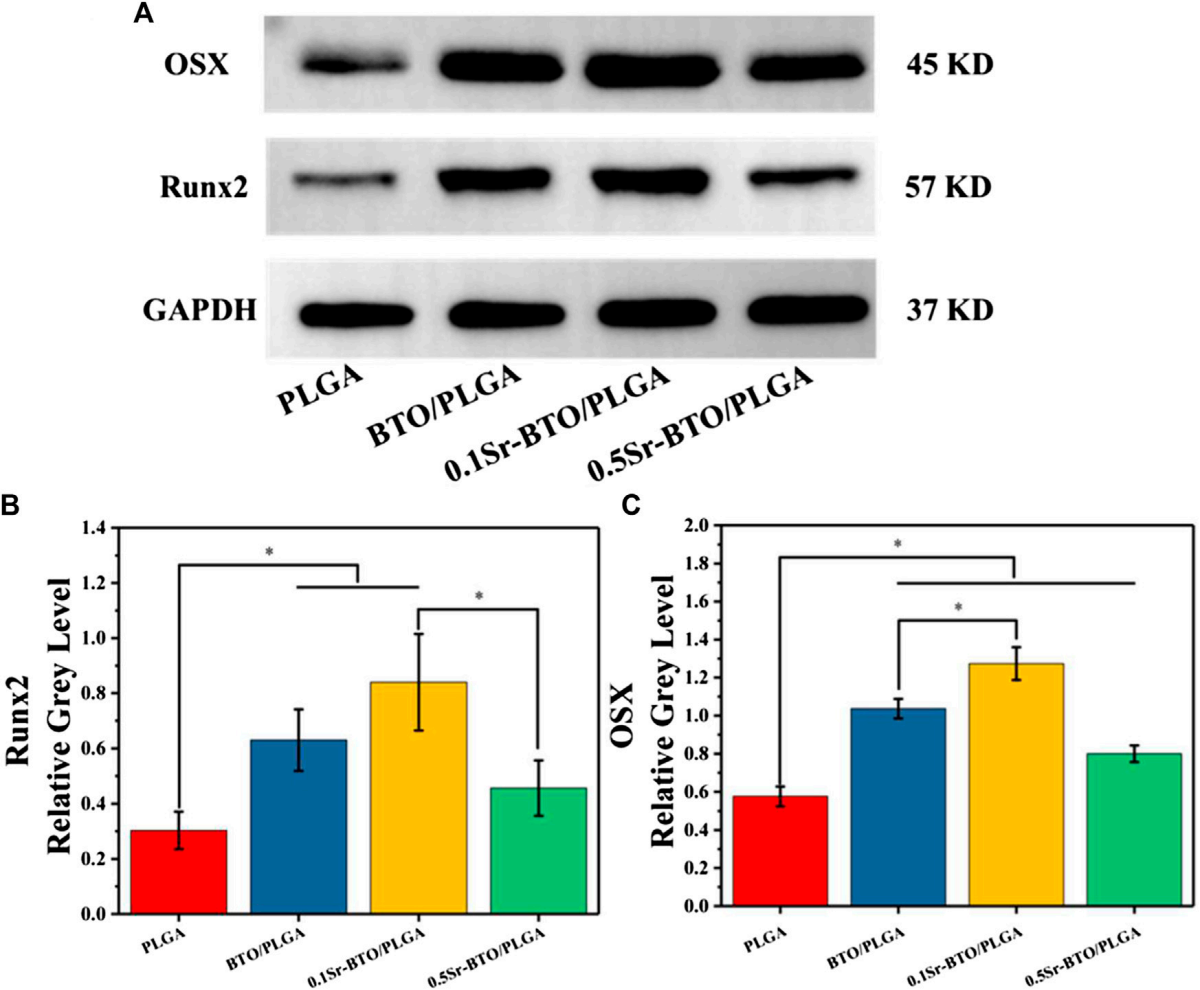
**(A)** Western blotting of Runx2 and OSX. **(B,C)** Quantitative analysis of, Runx2 and Osterix proteins.

The qRT-PCR assays reflecting osteogenic gene expressions, including runt-related transcription factor 2 (Runx2), Osterix (Osx), Osteopontin (OPN), and collagen type I (Col-1) were used to further evaluate the osteogenic differentiation ability of cells in microspheres (Figure 7). Runx2 is regarded as a key transcription factor that regulates the differentiation of cells into osteoblasts during bone development and it is also a sign of that osteoblasts begin to differentiate. OPN, an important bone matrix protein, is closely related to bone formation and development. As shown in Figures 7A—C, compared with the PLGA group the introduction of nanoparticles could significantly upregulate the Runx2 and OPN gene expressions (*p < 0.05). Especially, compared with the 0.5Sr-BTO/PLGA microsphere groups, the BTO/PLGA and 0.1Sr-BTO/PLGA microspheres were more favorable for promoting the expression of RUNX2, which indicated that the lower surface potential of the microspheres was better to promote the osteogenic differentiation. Moreover, Runx2 could improve the expression level of Osx, a downstream transcription factor of Runx2, which is a transcription factor related to osteoblast differentiation and bone formation (Yan et al., 2021). As summarized in Figure 7B, similar to the expression trend of RUNX2, BTO/PLGA and 0.1Sr-BTO/PLGA microspheres are beneficial to promote OSX gene expression due to their lower surface potential. Furthermore, Col-1 is the main component of collagen, whose increase in expression indicates that the extracellular matrix begins to deposit. As shown in Figure 7D, like the gene expression level of Runx2 and Osx, Col-1 were markedly upregulated in the BTO/PLGA and 0.1Sr-BTO/PLGA group, while no significantly differences were observed between PLGA and 0.5Sr-BTOPLGA. Furthermore, as shown in Figure 8, western blotting was conducted to further reveal the osteogenic ability through osteogenic-related proteins (Runx2 and OSX). Clearly, the protein levels of Runx2 and OSX were upregulated in BTO/PLGA and 0.1Sr-BTO/PLGA groups compared with PLGA and 0.5Sr-BTO/PLGA group, which indicated BTO/PLGA and 0.1Sr-BTO/PLGA could promote osteogenic ability. Moreover, the expression level of osteogenesis-related proteins in the 0.1Sr-BTO/PLGA group was higher than that in the BTO/PLGA group, indicating that the lower surface potential is more helpful to improve the osteogenic differentiation ability. Therefore, the change of the surface potential of the microspheres in this study is the main factor regulating the osteogenic differentiation of cells.

As mentioned above, through the comprehensive evaluation of ALP, ARS and the expression of osteogenic related genes and protein, the modulating surface potential of microspheres by introduction of nanoparticles could effectively regulate the osteogenic differentiation and ECM mineralization. Furthermore, the 0.1Sr-BTO/PLGA group exhibited an excellent ability to promote osteogenic differentiation due to its lower surface potential. Subsequently, the potential mechanism of Sr-BTO/PLGA microspheres promoting osteoblast differentiation was further explored.

### 3.4 Possible mechanism of osteogenic differentiation of MC3T3-E1 cells

These *in vitro* results suggested that the Sr-BTO/PLGA microspheres especially the 0.1Sr-BTO/PLGA microspheres contribute to promoting osteogenic differentiation. In this study, Sr^2+^ release and the electrical properties of microspheres were speculated to be the most possible factors affecting osteoblast differentiation. Firstly, strontium-containing pharmaceutical agent have been used in the clinical treatment of osteoporosis, because Sr^2+^ have the ability to promote the differentiation of osteoblasts and inhibit the activity of osteoclasts (Jia et al., 2017). However, the biological action of Sr^2+^ possess a dose dependent manner. Previous research has shown that when the Sr^2+^ concentration is in the range of 2-6 μg/ml, the relative cell viability is increased and has the effect of promoting osteogenic differentiation (Naruphontjirakul et al., 2018). As shown in the results related to osteogenic differentiation, BTO doped with higher strontium content (0.5Sr-BTO/PLGA group) did not significantly improve the effect of osteogenic differentiation, which may be due to the fact that the strong constraints of BTO lattice structure make them difficult decompose, corresponding resulting low Sr^2+^ release. According to the previous Sr^2+^ release results, when microspheres were soaked in PBS for 21 days (Figure 2D), the final release concentration of Sr^2+^ was only 0.23 ± 0.01 and 1.18 ± 0.02 μg/ml in the 0.1Sr-BTO/PLGA and 0.5Sr-BTO/PLGA group, respectively, which was lower than the effective concentration to perform the effect of promoting osteogenic differentiation. Therefore, the regulation of osteogenic differentiation by Sr^2+^ is not the main factor in this study.

**FIGURE 9:**
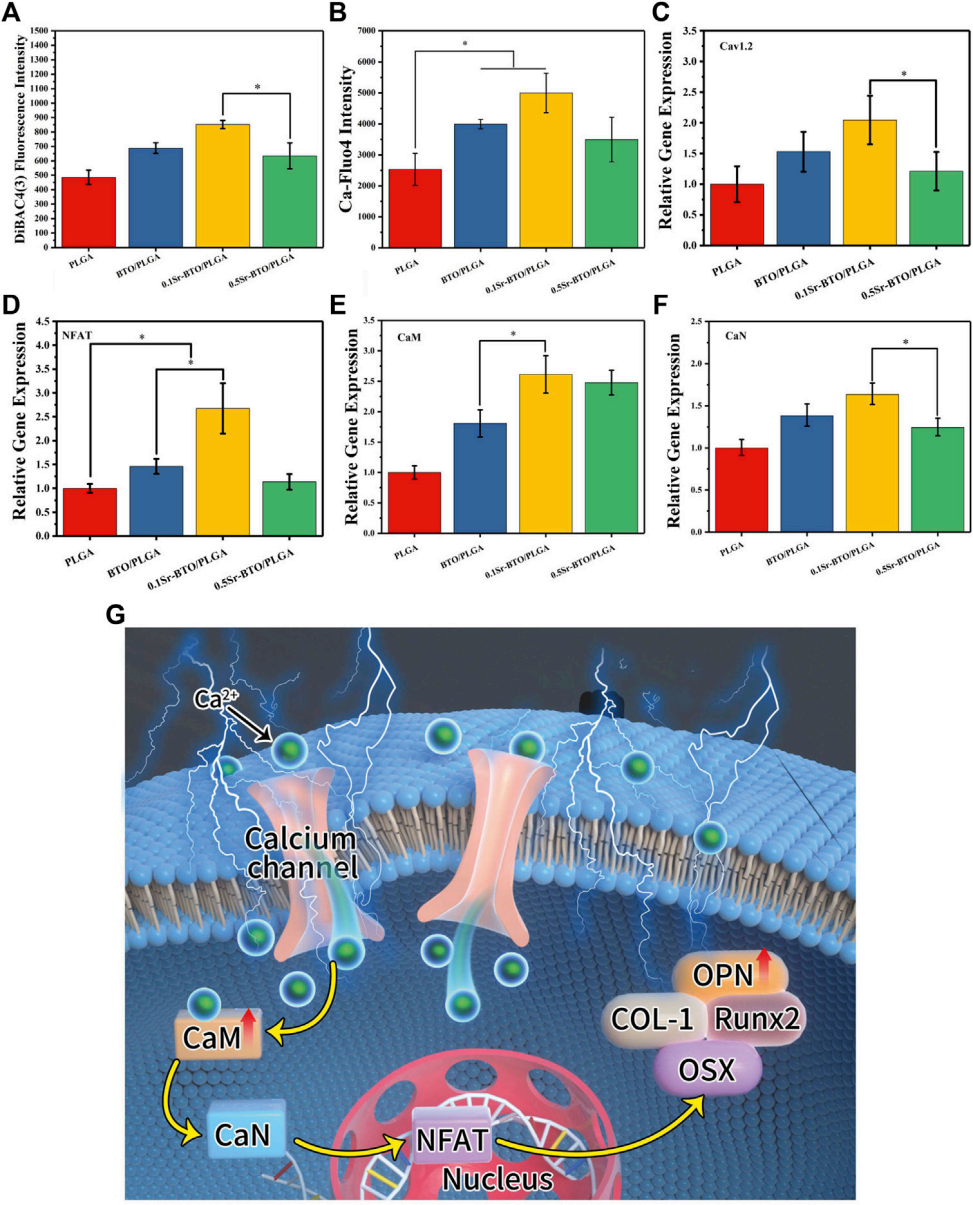
**(A,B)** Cell membrane potential and fluorescence intensity of the Ca-Fluo-4 complex for PLGA, BTO/PLGA and Sr-BTO/PLGA microspheres at 1 day. RT-PCR results of MC3T3-E1 cultured on different microspheres for 7 days: relative gene expression levels of **(C)** Cav 1.2, **(D)** NFAT, **(E)** CaM, **(F)** CaN; GADPH was used as a reference gene**.** *p < 0.05 **(G)** Schematic diagram of osteogenic differentiation of cell on microspheres.

After excluded the effect of Sr^2+^ release on osteogenic differentiation, the electrical properties play another pivotal role in regulating osteogenic differentiation. Previous studies have shown that electrical signals regulate osteogenic differentiation by activating calcium-sensing receptors, increasing calcium influx, and upregulating more cytosolic calcium ions (Yu et al., 2021). Furthermore, ion channels and pumps on cell membranes are activated, accompanied by changes in transmembrane potential, thereby guiding a battery of cell behaviors (e.g., adhesion, proliferation, differentiation) (Yu et al., 2021). To verify the cellular response to sensing the surface potential of the microspheres, the cell membrane potential, intracellular Ca^2+^ concentration and calcineurin-NFAT signaling related gene expression levels were evaluated. Firstly, the alteration of cell membrane potential was measured with DiBAC_4_(3) [(Bis-(1,3-dibutylbarbituric Acid] Trimethine Oxonol), which is a lipophile anion fluorescent dye used to detect the alteration of cell membrane potential (Fu et al., 2019). DiBAC_4_(3) fluoresces only after entering cells and binding with proteins in the cytoplasm. When DiBAC_4_(3) enters the cell, the intensity of intracellular fluorescence increases, that is, the increase in membrane potential indicates cell depolarization. The DiBAC_4_(3) fluorescence intensity of the 0.1Sr-BTO/PLGA microspheres was higher than that of the other groups, suggesting that cell sensed the stimulation of the lower surface potential on 0.1Sr-BTO/PLGA microspheres to adjust the cell membrane potential (Figure 9A). Membrane potential depolarization was contributed to capacitive coupling between the cell membrane and the surface potential of 0.1Sr-BTO/PLGA microspheres. Secondly, dynamic changes in membrane potential can activate voltage-gated calcium channels. As shown in Figure 9C, the up regulation of gene expression of L-type voltage–gated Ca^2+^ channel (Cav1.2) of 0.1Sr-BTO/PLGA microsphere was observed, which indicated that the change of cell membrane potential in 0.1Sr-BTO/PLGA microsphere treated group promoted the high expression of Cav1.2, which is a voltage-dependent ion channel that regulates free calcium concentration in living cells by opening or closing the channel or regulating the expression of the channel. Thirdly, it is reported that intracellular Ca^2+^ concentration oscillation and calcium-sensing receptor activation play a key regulatory role in the process of osteogenic differentiation (Winslow et al., 2006; Liu et al., 2017). As the second messenger in cells, intracellular Ca^2+^ play an important role in in the process of intracellular signal interaction. The concentration of intracellular Ca^2+^ is related to Ca^2+^ entering the cytoplasm via plasma membrane channels, such as the abovementioned voltage-gated calcium channels (Cav1.2). To investigate whether the lower surface potential of microspheres could increase intracellular Ca^2+^, Fluo-4 AM, (a Ca^2+^-sensitive fluorescent probe), was used to measure intracellular Ca^2+^ concentration. As shown in the Figure 9B, the result of Fluo-4 fluorescence intensity showed that the introduction of nanoparticles to microspheres promotes the increase of intracellular Ca^2+^ concentration. Since the negative surface potential of the 0.1Sr-BTO/PLGA microspheres was the lowest among different microspheres, the intracellular Ca^2+^ changes were the most obvious in the 0.1Sr-BTO/PLGA microspheres group. Finally, the gene expression of CaM (calmodulin), CaN (calcineurin) and NFAT (nuclear factor of activated T-cells), which are related to intracellular Ca^2+^, may provide more information on Sr-BTO/PLGA microspheres to regulate MC3T3-E1 cells differentiation. As reveled in Figures 9D–F, under the activation of intracellular Ca^2+^ by the negative surface potential, the representative CaM, CaN, and NFAT gene expression of MC3T3-E1 were upregulated. To further determine the role of Ca^2+^ as a second messenger in osteogenic differentiation, the calcium channel blocker verapamil was used to inhibit the calcium channels. As shown in Supplementary Figure S5C, following treatment with verapamil, 0.1Sr-BTO/PLGA group did not significant increase the intracellular Ca^2+^ in MC3T3-E1, which indicated that 0.1Sr-BTO/PLGA increase intracellular Ca^2+^ by calcium channel. Furthermore, we evaluated the effect of blocking calcium on MC3T3-E1 osteogenic differentiation. As shown in Supplementary Figures S5A,B, after blocking calcium channels, the activity of ALP was significantly reduced in 0.1Sr-BTO/PLGA group. Moreover, we also assessed the expression of osteogenesis-related genes (Runx2, Col-1, OPN, and OSX). As shown in Supplementary Figures S5D—G, after blocking calcium channels, the expression of osteogenesis-related genes was significantly down-regulated. The above results indicated that the lower negative surface potential of the microspheres activated the opening of calcium ion channels, and calcium ions played a key role as a second messenger in promoting osteogenic differentiation. Taken together, the basic working principle for Sr-BTO/PLGA microspheres *in vitro* is presumed in Figure 9G. Under the stimulation of the negative surface potential of the microspheres, the cells adjust the membrane surface potential to open Ca^2+^ channels and promote the increase of the intracellular Ca^2+^ concentration. Ca^2+^ acts as a second messenger to further regulate the downstream CaN/NFAT signaling pathway to promote osteogenic differentiation.

### 3.5 Bone regeneration evaluation *in vivo*

In light of osteogenic bioactivity *in vitro*, bone defect healing experiments *in vivo* is the most effective method and significant target to further evaluate the osteogenic functionality of bone graft material. Therefore, to assess the bone healing efficacy of Sr-BTO/PLGA microspheres, various microspheres were implanted to cover the circular critical size bone defect (diameter of 5 mm) on the SD rat skull for *in vivo* evaluation.

#### 3.5.1 Assessment of bone formation by MRI and micro-CT image

**FIGURE 10:**
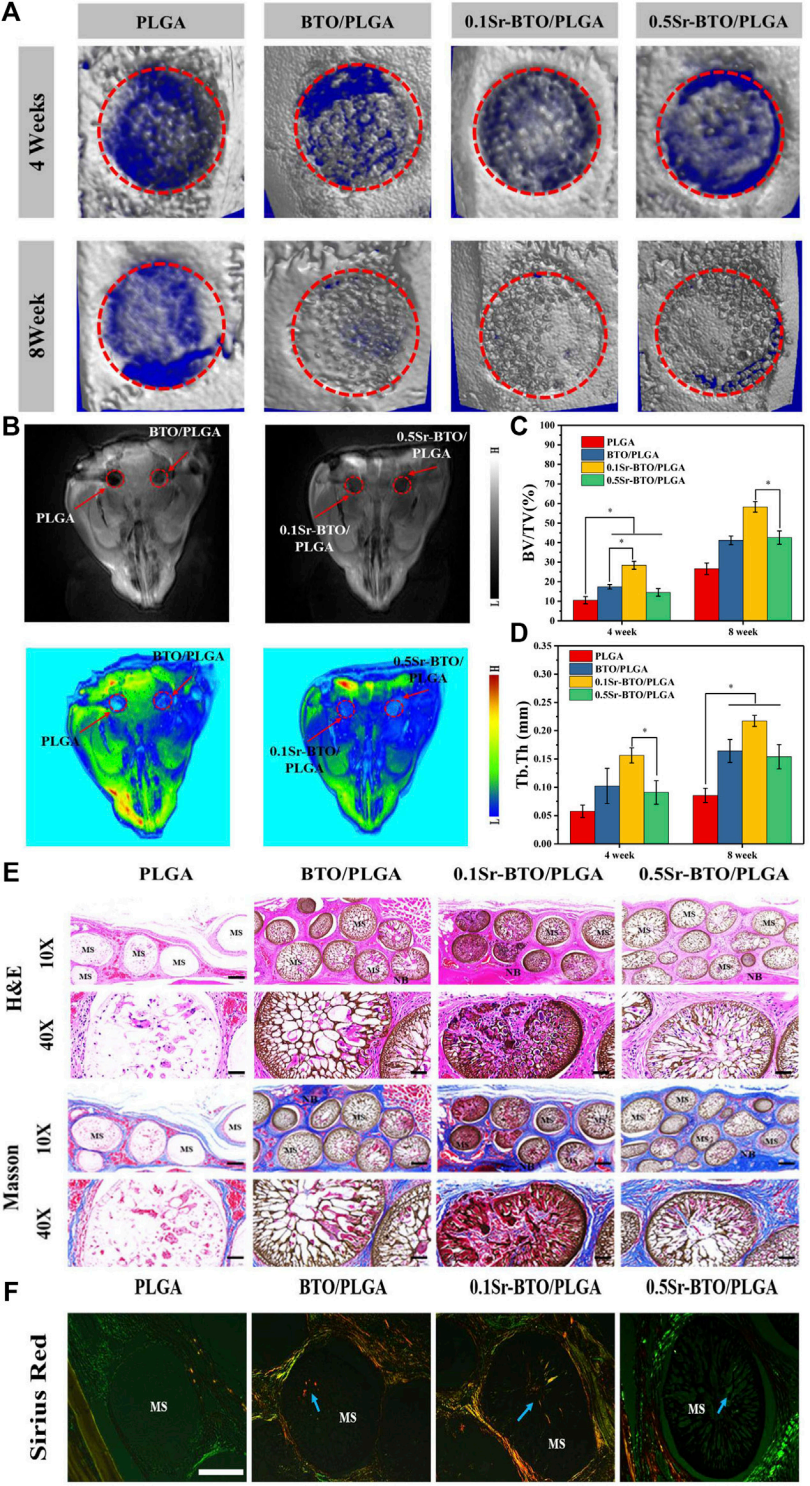
**(A)** 3D reconstructed micro-CT image of sagittal sections of bone formation at the rat calvarial bone defect areas due to implantation with different microspheres at 4 and 8 weeks. **(B)** *In vivo* T1-weighted grey-scale MRI image and the corresponding pseudo-color MRI image of bone formation at the rat calvarial bone defect areas due to the implantation with different samples at 8 weeks. **(C)** The quantitative micro-CT bone parameters of bone volume/tissue volume (BV/TV) ratio and **(D)** trabecular thickness (Tb.Th) at 4 and 8-weeks post-implantation in the experimental rat skull. *p < 0.05. **(E)** Histological analysis of Hematoxylin-Eosin **(H,E)** stained sections and Masson Trichrome staining in rat calvarial bone defect areas at 8 weeks post-surgery at 10x (Scale bar: 200 μm) and 40x (Scale bar: 50 μm) **(F)** Sirius Red staining of microsphere implanted in rat calvarial bone defect areas at 8 weeks post-surgery. (Scale bar: 200 μm).

The combined use of microcomputed tomography (micro-CT) and magnetic resonance imaging (MRI) could evaluate the bone repair effect of these microspheres more comprehensively. Initially, to quantitative investigate the bone regeneration efficiency of as-prepared microspheres, 3D reconstruction images of the calvarial defect area were obtained using micro-CT at 4- and 8-weeks post-operation. As evidenced by micro-CT images (Figure 10A), the degree of bone restoration occurring in the defect area filled with BTO/PLGA, 0.1Sr-BTO/PLGA and 0.5Sr-BTO/PLGA microspheres group was found to be more significantly higher than that of pure PLGA microspheres at 4 and 8 weeks. For all the microspheres contained nanoparticles groups, a substantial amount of new bone was formed and evenly distributed over the fracture, while the PLGA group was left with an obvious defect cavity. Specifically, much more new bone was evenly formed in the defect with 0.1Sr-BTO/PLGA microspheres. Moreover, the microarchitectural parameters of the newly formed bone within the cranial defect were quantified to understand the quality of the regenerated bone and confirm the above results. The ratio between the bone volume (BV) within the circular gap and the total volume (TV) within the circular gap (BV/TV) and trabecular thickness (Tb.Th) of each group as the major index were evaluated the quality of the regenerated bone. With increasing implantation time, the BV/TV and Tb.Th of the all group increased. As expected, the results showed that the BV/TV and Tb.Th in microspheres contained electroactive nanoparticles were significantly higher than that in the PLGA group. Specifically, the BV/TV in the defect area at 4 and 8 weeks of the 0.1Sr-BTO/PLGA group reached 28.44 ± 2.02 and 58.27 ± 2.69%, respectively, which was remarkably increased compared with that of other groups (Figure 10C). Similarly, the Tb.Th in the 0.1Sr-BTO/PLGA group (Figure 10D, 0.156 ± 0.013 mm for 4 weeks and 0.217 ± 0.009 mm for 8 weeks) was also the largest compared with that of other group. Therefore, the 0.1Sr-BTO/PLGA microspheres yielded significant enhancement of the bone regeneration capacity, which were attributed to the lowest surface potential. Although the *in vitro* 0.5Sr-BTO/PLGA group had a weaker pro-osteogenic effect than the BTO/PLGA group, no discernible difference was observed during the whole implantation period between the BTO/PLGA and 0.5Sr-BTO/PLGA groups, revealing that the Sr^2+^ in the 0.5Sr-BTO/PLGA group have a certain ability to enhance bone regeneration *in vivo*. Furthermore, *in vivo* T1-weighted greyscale MRI images and the corresponding pseudo-color MRI images of bone formation at the rat calvarial bone defect areas were also investigated at 8 weeks (Figure 10B). MRI appears to be a non-invasive and nonionizing technique well suited to *in vivo* longitudinal evaluation of tissue repair following a grafting procedure. The relaxation times depend on the stiffness of materials (Ravindran et al., 2015). As the stiffness of the material changes, the dipole coupling of protons changes and is reflected as a change in relaxation time. As shown in MRI images of the skull defect area, MRI signals were observed in the defect area, which may be due to the increased stiffness of the defect area caused by new bone formation at 8 weeks after surgery. Furthermore, a similar trend was drawn to the MRI results, the defects treated with BTO/PLGA, 0.1Sr-BTO/PLGA and 0.5Sr-BTO/PLGA microspheres showed significantly higher MRI signal enhancement, as compared to the PLGA microspheres. In addition, much more MRI signal was observed in the defect filled with 0.1Sr-BTO/PLGA microspheres, and the corresponding pseudo-color was closer to the surrounding mature bone tissue, suggesting that the 0.1Sr-BTO/PLGA microspheres may have a good ability to induce osteogenic differentiation *in vivo*. Combining the results of MRI and micro-CT, 0.1Sr-BTO/PLGA microspheres could greatly promote the regeneration of bone tissue, which may be because 0.1Sr-BTO/PLGA has unique electroactive properties.

#### 3.5.2 Histological analysis of bone regeneration

**FIGURE 11:**
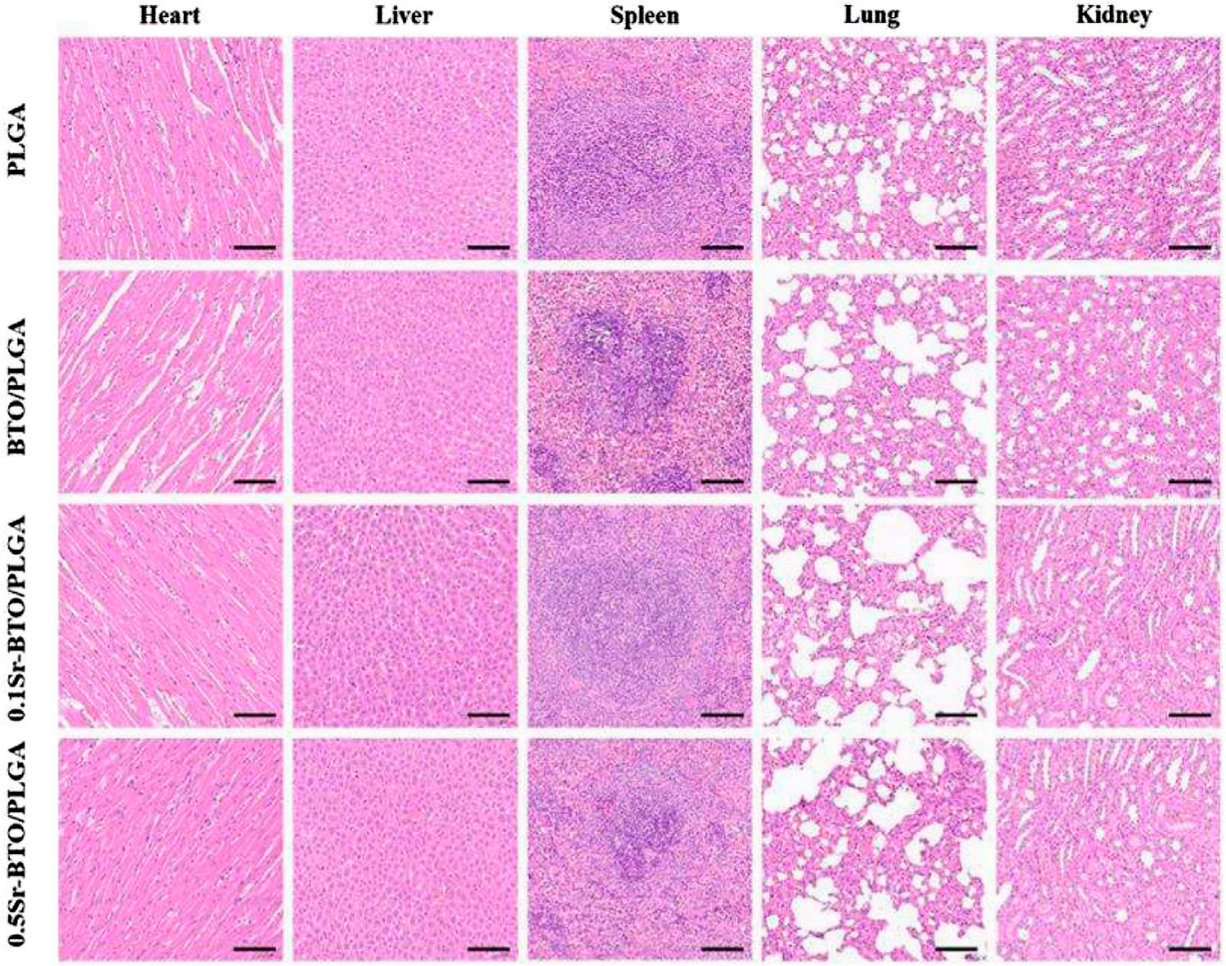
Biological assessment of lung, liver, spleen, kidney, and heart. The scale bar is 100 μm.

To further evaluate the efficacy of microspheres in accelerating the growth of collagen and new bone tissue when the microspheres were implanted into the defect site 8 weeks later, serial cross-sections of decalcified tissue were carried out by histological staining analysis (Hematoxylin and Eosin (H and E), Masson’s trichrome staining and picrosirius red staining). After 8 weeks of healing, histological analysis showed no obvious evidence of severe inflammatory responses in any of the specimens. In H and E staining (Figure 10E), the cell nuclei were stained blue, and the collagenous connective tissues and new bone matrix were highlighted in pink to varying degrees. In good agreement with micro-CT and MRI results, more collagen components were observed in the defects filled with BTO/PLGA and Sr-BTO/PLGA microspheres than in those filled with PLGA, which might be ascribed to the occurrence of osteogenesis. In addition, compared with PLGA microspheres, microspheres containing electroactive nanoparticles have a more obvious porous structure formation, accompanied by the growth of new tissues. Due to the formation of penetrating pores, the microspheres containing nanoparticles were filled with more cell and tissue matrix. Furthermore, the larger amount of mature dense bone tissue appeared in the BTO/PLGA and Sr-BTO/PLGA groups. Notably, the 0.1Sr-BTO/PLGA group was more attractive for new tissues to grow into the microspheres, reflecting that a large number of new bones formed that may arose from the lower surface potential of microspheres. Masson’s trichrome staining, as a specific staining method for collagen fibers, was further used to evaluate the formation and maturation of bone tissue. Likewise, Masson’s trichrome staining displayed that the collagen density was higher in the 0.1Sr-BTO/PLGA microsphere group than in the other three groups benefitting from the lower surface potential and pore formation. In addition, this observation is further consolidated by Picrosirius Red staining that reacts with collagen fibers to enhance their birefringence and resolution and shows different colors under polarized light (type I collagen appears strong orange and type III collagen appears green). As shown in Figure 10F, much more type 1 collagen fibers were found in the BTO/PLGA and 0.1Sr-BTO/PLGA group. For the 0.5Sr-BTO/PLGA group, although the osteogenic activities less than 0.1Sr-BTO/PLGA, it was higher than that of the PLGA microspheres. Moreover, the biosecurity of microsphere was further evaluated and shown in Figure 11. From the biological assessment of the main organs (lung, liver, spleen, kidney, and heart), all microspheres had no toxic effect on the main organs *in vivo*. These results indicate lower surface potential modulated by phase composition of electroactive nanoparticles in microsphere could effectively regulate bone regeneration, which provide a prospect for clinical treatment of critical-size bone defects.

## 4 Conclusion

In this work, Sr-BTO NPs with different content of tetragonal phase were successfully prepared by regulating the amount of Sr^2+^. Subsequently, Sr-BTO NPs functionalized microspheres with different surface potential were prepared with the aid of high-voltage electrostatic field. The surface potential of the microspheres can be effectively modulated by adjusting the phase composition of the inorganic component. Microspheres with suitable amount of Sr^2+^ doping can perform excellent osteogenic differentiation ability and enhance bone regeneration due to their lower surface potential. It is speculated that lower surface potential can induce Ca^2+^ influx and activate CaN/NFAT signaling pathways to promote osteogenic differentiation. Therefore, modulating the electric microenvironment of microspheres not only provide a promising strategy for promoting the repair of irregular bone defects, but also provides a prospective exploration for electrical stimulation therapy on tissue regeneration.

## Data Availability

The original contributions presented in the study are included in the article/Supplementary Material, further inquiries can be directed to the corresponding authors.
