# Supplementary material for: Modulating the surface potential of microspheres by phase transition in strontium doped barium titanate to restore the electric microenvironment for bone regeneration
Source: Front Bioeng Biotechnol. 2022 Aug 30;10:988300. doi: 10.3389/fbioe.2022.988300 (PMC9468715; doi:10.3389/fbioe.2022.988300)
Supplement: Supplementary file 1 [file DataSheet1.PDF]

Supporting information

Modulating the Surface Potential of Microspheres by Phase Transition in Strontium Doped Barium Titanate to Restore the Electric Microenvironment for Bone Regeneration

Peng Wang[a,b], Xiaosong Zhou[a,b], Caili Lv[a,b], Yu Wang[a], Zongliang Wang[a], Liqiang Wang[c], Yongzhan Zhu[d], Min Guo[a]*, Peibiao Zhang[a,b]*

[a] Key Laboratory of Polymer Ecomaterials, Changchun Institute of Applied Chemistry, Chinese Academy of Sciences, 5625 Renmin Street, Changchun 130022, P. R. China.

[b] School of Applied Chemistry and Engineering, University of Science and Technology of China, 96 Jinzhai Road, Hefei, Anhui 230026, P. R. China.

[c] Department of Ophthalmology, Third Medical Center, Chinese PLA General Hospital, Beijing 100853, China.

[d] 8th Department of Orthopaedics, Foshan Hospital of Traditional Chinese Medicine, Foshan 528000, P. R. China.

* Corresponding author's E-mail: zhangpb@ciac.ac.cn; guomin@ciac.ac.cn

Nanoparticles Toxicity Assessment

In order to evaluate the cytotoxicity of nanoparticles, using the extraction liquor of nanoparticles to cultivate MC3T3-E1 cell and measured by Cell Counting Kit-8 (CCK-8) was purchased from seven sea. The nanoparticles were immersed in culture medium to prepare a suspension (200 mg/mL). The suspension was placed in a shaker at 37 °C and oscillated at 100 rpm for 24 h. The MC3T3-E1 cells were cultured in 96 well tissue culture plate (Costar, 1×104 cells in 100 μL per well) with DMEM for 24 h (5% CO2, 37°C). Subsequently, the culture medium was replaced with nanoparticle extraction liquor diluted with gradient. Then the cells were cultured for 24 hours under the same conditions. Discard the extraction liquor to add 100 μL of medium and 10 μL CCK-8 per well, incubated for another 2 h under the same conditions. Finally, the absorbance at 450 nm was measured by multifunctional microplate scanner (Infinite M200, Tecan, Switzerland). In addition, the viability of cells exposed to nanoparticles for 24 h was also measured to evaluate the material cytotoxicity. Nanoparticles were dispersed in culture medium by ultrasonication for several hours to form nanoparticles dispersions (62.5 μg/mL). These dispersions were used in cell experiments, as described above. The morphology of osteoblasts treated with nanoparticles was observed under a light microscope.

Cell adhesion and Proliferation Assays

Cell attachment was investigated using Alexa-Fluor 594 phalloidin and 4, 6-diamidino-2-phenylindole (DAPI) staining. These microspheres were soaked in 75% ethanol solution and sterilized by ultraviolet for 1 days. After the ethanol on the surface of materials was washed away with PBS, these microspheres were placed into each well of a 24-well plate (Costar). MC3T3-E1(5×10^4 cells/well) were seeded onto microspheres and incubated in a humidified atmosphere with 5% CO_2 at 37 °C. After 3 day of culture, the cells attached to microspheres were fixed with 4% paraformaldehyde and then stained with DAPI and phalloidin. The images were captured by a fluorescent inverted microscope (TE2000U, Nikon). The spreading areas of cells on microspheres were measured using image J software (National Institutes of Health, Bethesda, MD, USA).

Live/dead staining was performed on cell/microsphere complexes by staining with calcein-AM/PI after 7 days of culture. Briefly, using Calcein-AM/PI treated cells for 10 min at 37 °C, and washed with sterile PBS three times. After staining was completed, samples were visualized by the fluorescent Inverted microscope. The proliferation assay of the MC3T3-E1 cells on various microspheres was evaluated via counting kit-8 (CCK-8) test. MC3T3-E1 (2×10^4 cells/well) were seeded onto microspheres in 24 well plate and incubated in a humidified atmosphere with 5% CO_2 at 37 °C. After 1, 4 and 7 days of culture, the medium was replaced by medium with 10% CCK-8. After 2h of incubation, 100 μL medium were transferred to 96-well cell culture cluster and measured by multifunctional microplate scanner at 450 nm.

Osteogenic Differentiation Assay

Alkaline phosphatase (ALP) staining was applied to assess the activity of ALP in MC3T3-E1 cells grown on different microspheres. Furthermore, to inhibit the L-type calcium channels, cells were treated with 10 μg/ml of the L-type calcium channel blocker verapamil (Sigma-Aldrich, St. Louis, MO). MC3T3-E1 (2×10^4 cells/well) were seeded onto microspheres in 24 well plate and incubated in a humidified atmosphere with 5% CO_2 at 37 °C. When cultured on different samples for 7 days, the cells were washed three times with PBS, fixed with 4% PFA for 15 min, and washed with PBS again. Thereafter, the cells were immersed in 500 μL ALP dye for at least 12 h at room temperature under dark conditions. The extra ALP dye was washed away with PBS and then the purple color intensity was observed by stereoscopic microscope. The ALP relative activity was tested by Alkaline phosphatase detection kit (Beyotime, China). After cultured on different microspheres for 7 days, MC3T3-E1 cells were washed with PBS three times and split by adding 200 mL of RIPA cell lysis solution, freezing at -80 ºC for 25 min and thawing at 37 ºC. Then, p-nitrophenol phosphate substrate and BCA solution were added, followed by incubation in the dark for 30 min at 37 ºC. The absorbances at 405 nm (OD_{405}) and 562 nm (OD_{562}) were read using a multifunction microplate scanner. The corresponding ALP quantitative evaluation was calculated according to the equation OD_{405}/OD_{562}.

The alizarin red staining was used to evaluate calcium-rich deposits in MC3T3-E1 cells grown on different microspheres. MC3T3-E1 (2×10^4 cells/well) were seeded onto microspheres in 24 well plate and incubated in a humidified atmosphere with 5% CO_2 at 37 °C. After the MC3T3-E1 on microspheres were continually cultured for 14 days. The cells were washed with PBS for 3 times and fixed with 4% paraformaldehyde for 15 min. Then the cells were stained in 1% (w/v) Alizarin Red S (ARS, Sigma) solution (1 g of ARS dissolved in 100 mL PBS, pH adjusted to 4.1-4.3) for 1 h at room temperature. After washed with PBS for 3 times, the presence of mineral deposition was qualitatively evaluated according to the red color intensity observed by stereoscopic microscope. Calcium quantification was tested by cetylpyridinium chloride (CPC). ARS-stained microspheres were washed with deionized water and subsequently treated with 1 ml 10% CPC solution for 1 h to desorb calcium ions. Absorbance was read at 540 nm in a multifunctional microplate scanner. The evaluation method of ALP and ARS of composite films is the same as that of microspheres.

Measurement of Intracellular Ca^{2+}

The intracellular Ca^{2+} levels were evaluated by Fluo4-AM calcium indicator (Beyotime, China). Furthermore, to inhibit the L-type calcium channels, cells were treated with 10 μg/ml of the L-type calcium channel blocker verapamil (Sigma-Aldrich, St. Louis, MO). MC3T3-E1 (2×10^4 cells/well) were seeded onto microspheres in 24 well plate and incubated in a humidified atmosphere with 5% CO_2 at 37 °C. MC3T3-E1 cells cultured on various microspheres were incubated for 1 day. Fluo4-AM dye loading solution (2μM in PBS) with 300μL was quickly but carefully added to each well and the plates were incubated at 37°C for 30 min. Then wash three times with PBS, add 300 μl of PBS and continue incubation at 37 °C for 30 minutes. After washing with PBS, cells were collected by 0.5mL trypsin. After three times of centrifugation, mean fluorescence intensity of cell suspension was measured by multifunctional microplate scanner (Tecan Infinite M200) with setting the exaction wavelength at 488 nm and emission wavelength at 518 nm.

Microspheres Activation of Membrane Potential Depolarization

24-well plates (10^5 cells/well) with microsphere were used to assess cell membrane potential. After culturing for 1 day, the cell culture medium was removed and DiBAC4(3) (2 μM) was added to culture the cells for 30 min at 37 °C. Evaluation of intracellular fluorescence intensity of stimulated cells with microplate reader (Excitation Wavelength 493 nm, Emission Wavelength 516 nm).

Quantitative Real-time Polymerase Chain Reaction (PCR)

The expression of the genes of calcineurin/NFAT signaling pathway and osteogenesis-related genes was quantitatively evaluated via real-time PCR. Furthermore, to inhibit the L-type calcium channels, cells were treated with 10 μg/mL of the L-type calcium channel blocker verapamil (Sigma-Aldrich, St. Louis, MO). MC3T3-E1 (2×10^4 cells/well) were seeded onto microspheres in 24 well plate and incubated in a humidified atmosphere with 5% CO_2 at 37 °C. After MC3T3-E1 cells cultured on various microspheres for 7 days, the total RNA of the cells cultured on different microspheres was extracted using TRIzol Reagent (Invitrogen, Thermo Fisher, USA) on the basis of the manufacturer's manual. The purity and concentration of RNA were assessed by Nanodrop Plates (Infinite M200, Tecan, Switzerland). The mRNAs of all the samples were reversely transcribed according to the description in Prime Script RT Reagent Kit with gDNA Eraser RR047A (TaKaRa, Japan). The expression of related genes was quantified using SYBR Premix Ex Taq RR420A (TaKaRa, Japan). Gene-specific primers containing glyceraldehyde-3-phosphate dehydrogenase (GAPDH), runt-related gene 2 (Runx2), osteopontin (OPN), Osterix (Osx) collagen type 1 (Col-1), calmodulin (CaM), Calcineurin (CaN), nuclear factor of active T-cells (NFAT), and L-type voltage–gated Ca^{2+} channel (Cav1.2) were designed by the primer design software of beacon 5.0. Real-time PCR analysis was implemented using Stratagene Mx3005P Real-time PCR System (Agilent Technologies Inc., USA) and the gene expression levels were acquired by the threshold cycles (Ct). Relative transcript quantities were calculated through using the ΔΔCt method. GAPDH was used as a reference gene and was amplified as well as the target genes from the same cDNA samples. The difference of the Ct value between the

sample and GAPDH was defined as the ΔCt. The difference in the ΔCt of the cells grown on the experimental groups relative to the control group cells was defined as the ΔΔCt. The fold change in gene expression was expressed as $2^{-\Delta\Delta Ct}$.

Western blot analysis

Western blot analysis was conducted to evaluate the expressions of Runx2 and OSX proteins. After 7 days of osteoinductive culture, cell/microsphere complexes were collected for lysis by adding RIPA Lysis Buffer (Applygen, China) supplemented with 0.5 mM phenylmethanesulfonyl fluoride (PMSF, Sigma, USA). The total protein was quantified using BCA protein assay Kit. Then, ~20 mg of total protein from cell lysate was loaded onto 8% sodium dodecyl sulfonate (SDS)-containing polyacrylamide (SDS-PAGE) gel (Solarbio, China), and further blotted onto poly(vinylidene difluoride) (PVDF) membrane (Immobilon P, Millipore). After being incubated with a blocking buffer containing 5% skim milk powder in TBST (Tris buffered saline with Tween) for 2 h, samples were further incubated with primary antibodies at 4 °C overnight, followed by being rinsed for 3 × 15 min. Then the samples were incubated with secondary antibody of HRP conjugated goat anti-Mouse or HRP conjugated goat anti-Rabbit IgG (1:5000, ab205718, Abcam, UK) at R.T. for 1.5 h. Finally, the samples were rinsed with TBST for 3 times and the signals were detected by Molecular Imager Versa Doc MP 4000 System (BioRad, USA). Band intensity was normalized. The primary antibodies used in this immunoblotting assay included rabbit anti-Runx2 antibody (1:500, ab6285, Abcam, UK), rabbit anti-OSX (1:1000, ab8448, Abcam, UK) and mouse anti-GAPDH antibody (1:5000, ab8245, Abcam, UK)

In vivo evaluations based on rat calvarial defect model

In a typical surgical procedure for creating rat calvarial defects, the rats were anesthetized using a subcutaneous injection of pentobarbital (Nembutal). The hairs on the cranium dorsum were shaved and handled aseptically for surgery. A sagittal incision of ~ 20 mm was operated on the scalp of rat to expose calvarium periosteum. Based on lambdoidal and sagittal sutures as landmarks, two full-thickness calvarial defects (5-mm diameter for each) were generated using a slow-speed dental drill

under continuous irrigation with 0.9% saline solution to prevent the local overheating. Microspheres were then implanted into the defect. Afterward, the incision was sutured by using 4.0 resorbable silk sutures. Each rat received a post-surgery antibiotic treatment for 5 d (applied amount of penicillin: 400000 IU/d). All rats were housed with a standardized diet in a room-temperature environment and monitored daily for potential complications or abnormal behaviors.

Samples	(Ba+Sr)/Ti	Molecular formula in theory	Molecular formula ICP Calculated	(Ba+Sr)/Ti ICP Calculated
0.5Sr-BTO	(7.5+0.5)/8	$Ba_{0.94}Sr_{0.06}TiO_3$	$Ba_{0.93}Sr_{0.07}TiO_3$	(7.44+0.56)/8
0.1Sr-BTO	(7.9+0.1)/8	$Ba_{0.99}Sr_{0.01}TiO_3$	$Ba_{0.98}Sr_{0.02}TiO_3$	(7.84+0.16)/8

Table S1. The molar ratio of Ba^{2+} to Sr^{2+} determined by ICP

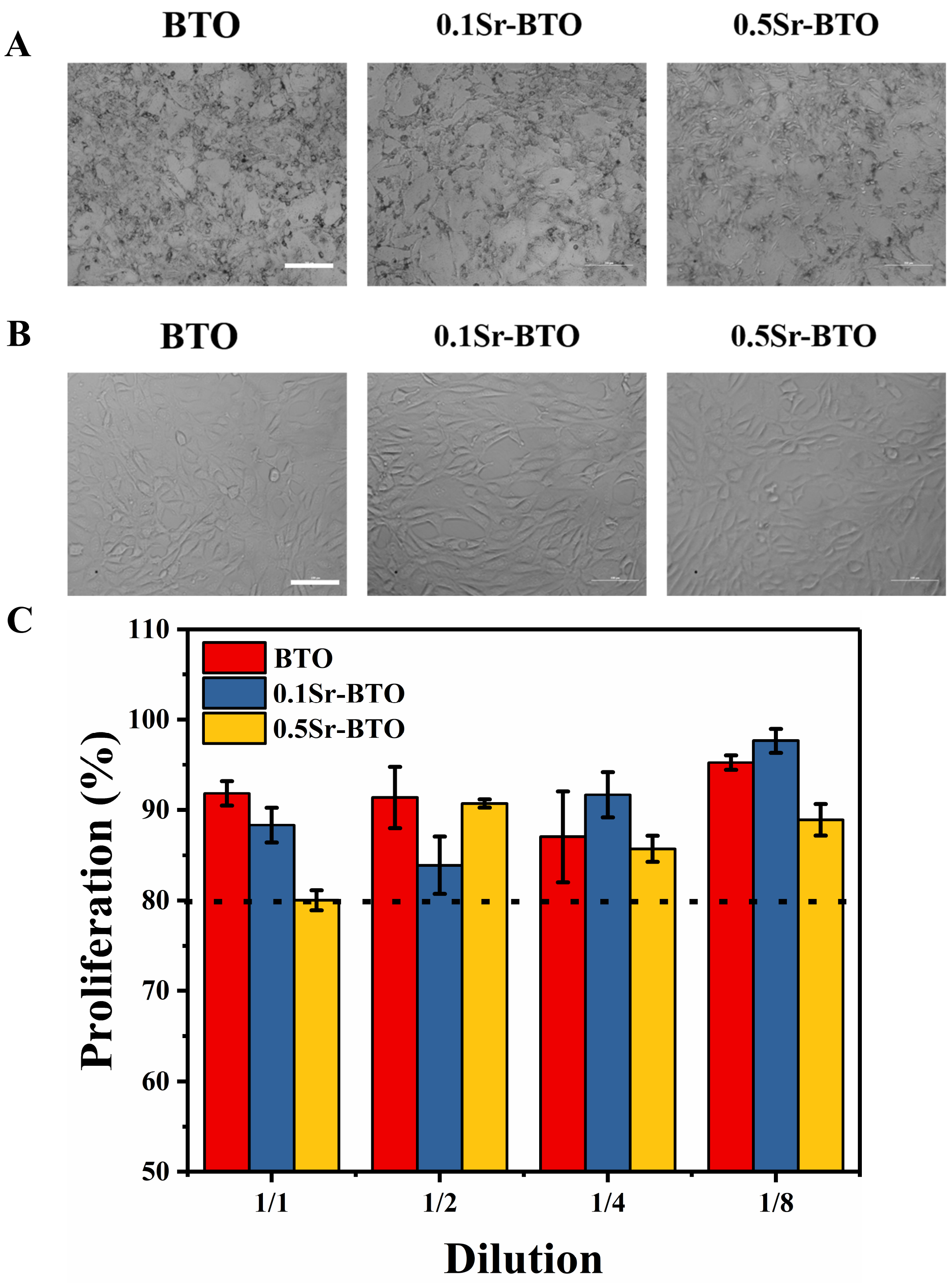

Figure S1. (A) Light microscopy images of osteoblasts incubated with nanoparticles at a concentration of 62.5 μg/ml. Scale bar: 200 μm. (B) Light microscopy images of osteoblasts cultured in the presence of different particles' original leaching liquor for 24 h. Scale bar: 100 μm. (C) Cytotoxicity of nanoparticles' leaching liquor on MC3T3-E1 cells detected by the CCK-8 assay.

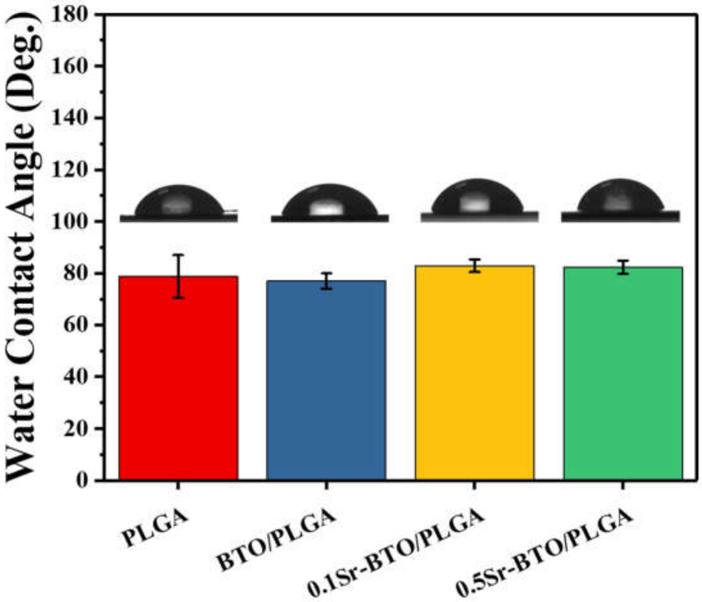

Figure S2. The contact angle of PLGA, BTO/PLGA, 0.1Sr-BTO/PLGA, and 0.5Sr-BTO/PLGA.

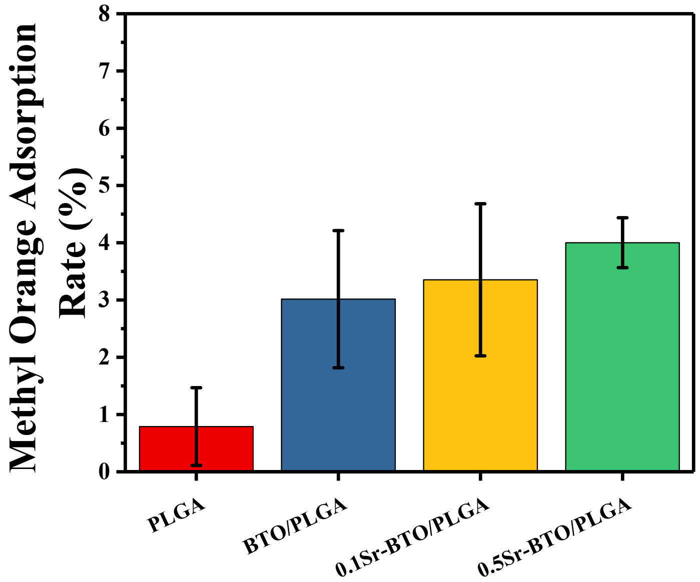

Figure S3. Adsorption rate of Sr-BTO/PLGA microspheres for methyl orange (MO) dyes.

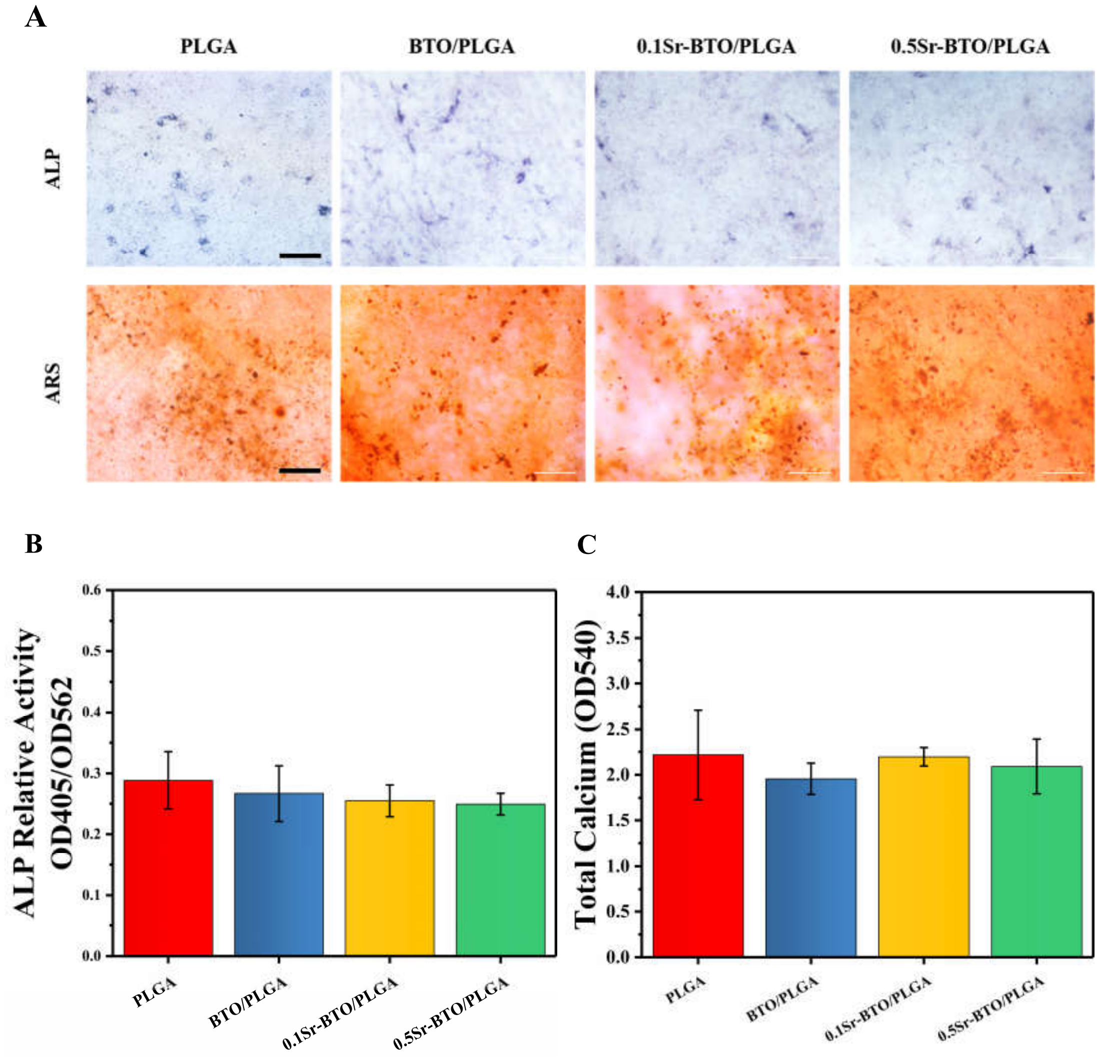

Figure S4. ALP staining and the corresponding quantitative evaluation of ALP relative activity in MC3T3-E1 cells cultured for 7 days on composite membrane (A&B). Alizarin Red staining and the corresponding quantitative evaluation of calcium content mineral deposition in MC3T3-E1 cells cultured for 14 days on different substrates (A&C). Scale bar: 200 µm, $*p < 0.05$.

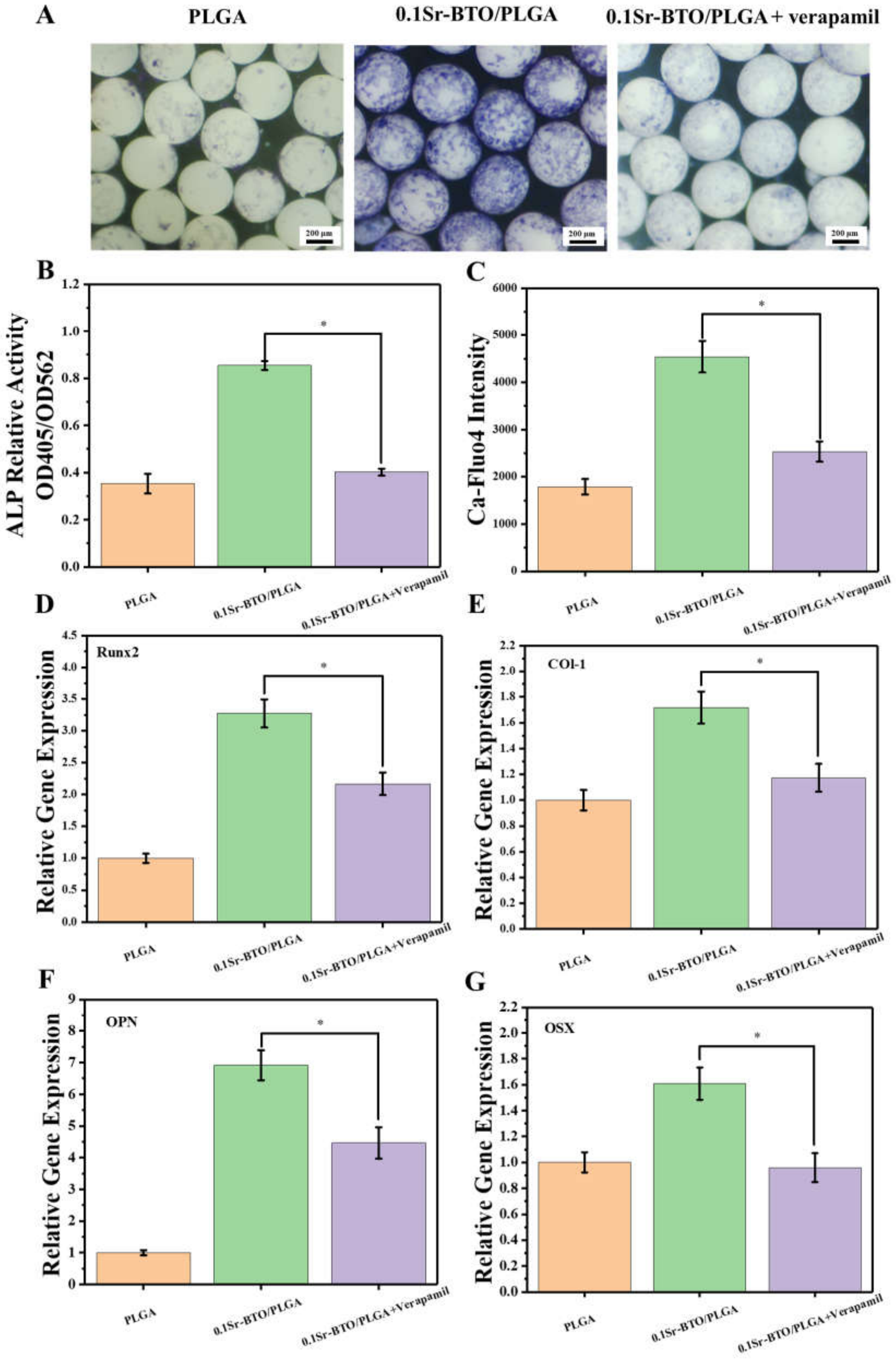

Figure S5. (A) The ALP staining of MC3T3-E1 cells cultured on microspheres. Scale bar: 200 μm (B) The quantitative evaluation of ALP relative activity of MC3T3-E1 cells cultured on microspheres for 7 days. (C) Fluorescence intensity of the Ca-Fluo-4

complex for microspheres. (D-G) RT-PCR results of MC3T3-E1 cultured on different microspheres for 7 days: relative gene expression levels of (D) Runx 2, (E) Col-1, (F) OPN, and (G) OSX; GADPH was used as a reference gene, $*p < 0.05$.
